# Anti-atherosclerotic effects of *Allium hookeri* and its mechanism in high fat/high cholesterol-induced obese C57BL/6 mice

**DOI:** 10.29219/fnr.v70.14716

**Published:** 2026-10-30

**Authors:** Seung-Hui Choi, Ha-Rin Moon, Daeun Hong, Jung-Mi Yun

**Affiliations:** 1 Department of Food and Nutrition, Chonnam National University, Gwangju, South Korea; 2 Global Convergence Research Division, World Institute of Kimchi, Gwangju, South Korea

**Keywords:** atherosclerosis, *Allium hookeri*, lipid metabolism, histone acetylation, regulatory T cells

## Abstract

**Background:**

Atherosclerosis is a chronic inflammatory disease characterized by plaque accumulation and narrowing of blood vessels. Dietary substances may help to prevent atherosclerosis by modulating lipid metabolism and inflammation. *Allium hookeri* (*A. hookeri*), a medicinal herb used in Southeast Asia, exhibits antioxidant and antidiabetic activities.

**Objective:**

This study evaluated the protective effects of *A. hookeri* water extract (AHWE) against atherosclerosis in C57BL/6 mice fed a high-fat/high-cholesterol diet (HFCD) (60% kcal from fat plus 1% cholesterol; HFCD) for 13 weeks.

**Design:**

Four-week-old male C57BL/6 mice were divided into four groups (*n* = 10/group): control (CON), HFCD, HFCD + AHWE 300 mg/kg/day, and HFCD + AHWE 500 mg/kg/day. Following 12 weeks of dietary intervention, the mice were sacrificed at 17 weeks of age. Body and organ weights, serum parameters, histological changes, and the expression of proteins and genes involved in lipid metabolism and inflammation were analyzed. The immunomodulatory effects of AHWE were evaluated using regulatory T cells (Tregs).

**Results:**

AHWE supplementation at 500 mg/kg/day reduced body weight gain, liver and adipose tissue weights, and serum levels of alanine aminotransferase, aspartate aminotransferase, triglycerides, total cholesterol, low-density lipoprotein cholesterol, and insulin, while increasing high-density lipoprotein cholesterol levels and reducing the atherogenic index in HFCD. AHWE alleviated hepatic lipid accumulation and aortic intimal thickening. Mechanistically, AHWE upregulated peroxisome proliferator-activated receptor gamma, liver X receptor alpha, and ATP-binding cassette transporter A1 expression and downregulated CD36, SR-A1, and LOX-1. In addition, AHWE promoted AMP-activated protein kinase activation, inhibited mechanistic target of rapamycin phosphorylation, and suppressed inflammatory responses by reducing nuclear factor-kappa B, cyclooxygenase-2, tumor necrosis factor-alpha, and interleukin-6 expression. AHWE increased Treg populations, and IL-10 secretion, while ex vivo analysis confirmed its anti-inflammatory effects. Moreover, AHWE inhibited H3K9 and H3K27 acetylation.

**Conclusion:**

These findings suggest that AHWE at 500 mg/kg/day may alleviate atherosclerosis by regulating lipid metabolism, inflammation, Treg-mediated immune balance, and histone acetylation.

## Popular scientific summary

- AHWE improved metabolic parameters and reduced aortic intimal thickening in HFCD mice
- AHWE promoted cholesterol efflux and reduced cholesterol uptake in HFCD-fed mice.
- AHWE attenuated inflammation by downregulating pro-inflammatory cytokines and enhancing Tregs immunoregulatory function.
- AHWE alleviated atherosclerosis by modulating histone H3K9 and H3K27 acetylation.

Atherosclerosis is a major contributor to cardiovascular diseases (CVDs) and is characterized by lipid accumulation and chronic inflammation within the arterial wall (1). Considering that atherosclerosis is a progressive condition that worsens with age, early intervention targeting modifiable risk factors – such as lipid levels and inflammatory responses – is essential (2). Persistently elevated plasma lipid levels enhance oxidative stress and impair endothelial integrity, facilitating low-density lipoprotein (LDL) oxidation and foam cell generation through disrupted cholesterol handling (3). The liver plays a central role in lipid homeostasis by regulating lipoprotein metabolism and reverse cholesterol transport, which removes excess cholesterol from peripheral tissues (4). Therefore, regulation of lipid metabolism and inflammatory responses is considered an important strategy for the prevention of atherosclerosis.

Disturbance of the balance between cholesterol influx and efflux promotes hepatic lipid deposition and accelerates metabolic abnormalities associated with CVD (5). Scavenger receptors, including SR-A1, CD36, and LOX-1, promote lipid accumulation through the uptake of oxidized low-density lipoprotein (ox-LDL) and other modified lipids (6, 7). In contrast, cholesterol efflux mediated by ATP-binding cassette transporter A1 (ABCA1) functions as a key cholesterol exporter that removes excess intracellular cholesterol, thereby preserving lipid homeostasis (7). In a study by Tarling et al., ABCA1 is induced by peroxisome proliferator-activated receptor gamma (PPARγ) and liver X receptor alpha (LXR-α) (8).

The AMP-activated protein kinase (AMPK)/mechanistic target of rapamycin (mTOR) pathway plays a central role in regulating lipid metabolism (9). AMPK activation exerts anti-atherogenic effects by limiting lipid synthesis and accumulation, reducing oxidative stress and inflammation, and improving endothelial function (9). In contrast, chronic inflammatory conditions, such as atherosclerosis and obesity, are associated with impaired AMPK signaling, which contributes to metabolic dysregulation and disease progression (10). Moreover, AMPK activation negatively regulates nuclear factor-kappa B (NF-κB) signaling, thereby reducing the transcription of pro-inflammatory cytokines and alleviating inflammation (11). Inflammation plays a central role in the initiation and progression of atherosclerosis, from endothelial dysfunction to plaque rupture (12). ox-LDL activates NF-κB signaling, which promotes the production of pro-inflammatory cytokines, including tumor necrosis factor-alpha (TNF-α), cyclooxygenase-2 (COX-2), and interleukin-6 (IL-6), thereby accelerating plaque formation (13). In addition, High-fat diet (HFD) has been shown to significantly increase the secretion of pro-inflammatory cytokines, such as IL-1β, IL-6, and TNF-α, compared with a standard diet (14).

Atherosclerosis is a chronic inflammatory disease initiated by innate immune responses and further shaped by adaptive immune mechanisms (15). As atherosclerosis progresses, adaptive immune responses are activated, involving CD4^+^ T cells that differentiate into T helper cell types 1 and 17 (Th1 and Th17) and regulatory T (Treg) cells (15). Pro-inflammatory Th cell subsets, including Th1 and Th17, promote inflammation through the secretion of cytokines such as interferon-gamma (IFN-γ) and IL-17. Conversely, Treg cells, characterized by the expression of the transcription factor Foxp3 and the IL-2 receptor CD25, exert protective effects by releasing anti-inflammatory cytokines, including IL-10 and transforming growth factor-beta (TGF-β), thereby suppressing excessive immune activation and mitigating atherosclerosis development (15). Accumulating evidence indicates that both the reduced numbers and dysfunction of Tregs contribute to the pathogenesis of atherosclerosis (16). Tregs exert anti-atherogenic effects primarily through the secretion of anti-inflammatory cytokines such as IL-10 and TGF-β, which collectively suppress the recruitment and activation of inflammatory cells, inhibit effector T cell differentiation, and attenuate vascular inflammation (17). Therefore, restoration of immune homeostasis through regulation of Treg function may contribute to the improvement of atherosclerosis (18).

Both DNA methylation and histone modifications are epigenetic changes relevant to atherosclerosis and recent studies have shown that these epigenetic regulatory systems are closely interrelated (19). Histone acetylation is a well-characterized epigenetic modification associated with transcriptional activation through chromatin relaxation and enhanced gene expression (19). In atherosclerosis, elevated levels of histone 3 lysine 9 acetylation (H3K9ac) and histone 3 lysine 27 acetylation (H3K27ac) are associated with the upregulation of genes involved in inflammation, lipid metabolism, cell adhesion, and endothelial dysfunction (20). Increased H3K27ac has been linked to enhanced expression of scavenger receptors, including CD36 and SR-A1, as well as pro-inflammatory cytokines such as TNF-α and IL-6, thereby promoting lipid accumulation and chronic vascular inflammation (21, 22). In addition, increased H3K9ac transcriptionally activates vascular cell adhesion molecule-1 (VCAM-1) and intercellular adhesion molecule-1 (ICAM-1), facilitating immune cell infiltration into the arterial wall during early lesion development (22).

Although lipid-lowering and anti-inflammatory agents are widely used for the prevention and treatment of atherosclerosis, long-term pharmacological therapy may be associated with potential adverse effects, including hepatic dysfunction, disturbances in glucose metabolism, and other treatment-related complications (1). Therefore, increasing attention has been directed toward natural bioactive compounds as potentially safer complementary strategies for the prevention and management of atherosclerosis (23). Nutritional interventions targeting lipid metabolism, inflammation, Treg regulation, and histone acetylation can be a safe, useful, and promising strategy for the prevention of atherosclerosis. Recent studies have shown that natural compounds, including polyphenols, alkaloids, and sulfur-containing compounds, exert anti-atherosclerotic effects by promoting cholesterol efflux and modulating inflammatory responses (23). In our previous study, phenethyl isothiocyanate (PEITC), a representative sulfur-containing compound, alleviated obesity-associated atherosclerosis by regulating lipid metabolism, inflammatory responses, and histone acetylation (24). *Allium hookeri* (*A. hookeri*), a plant native to Southeast Asia traditionally used as a food ingredient and folk medicine, contains various bioactive compounds, including saponins and sulfur-containing amino acids such as cycloalliin (25, 26). Recent studies have reported various biological activities of *A. hookeri* roots, including anti-inflammatory, anti-obesity, and immunomodulatory effects (27–29). However, the potential protective effects of *A. hookeri* against atherosclerosis have not been extensively investigated. This study aimed to investigate the anti-atherosclerotic effects of *A. hookeri* water extract (AHWE) in C57BL/6 mice fed a high-fat/high-cholesterol diet (HFCD), focusing on regulating lipid metabolism disorders, suppressing inflammatory responses, enhancing the population and immunoregulatory function of Tregs, and reducing histone acetylation.

## Materials and methods

### Materials

*Allium hookeri* root powder was sourced from an online marketplace (Gmarket, Seoul, Korea) and manufactured by Nokshipcho Co., Ltd. (Shinan-gun, Jeollanam-do, Korea). Antibodies against SR-A1, CD36, ABCA1, and Foxp3 were sourced from Abcam (Cambridge, UK), while antibodies targeting LOX-1, LXRα, PPARγ, TNF-α, COX-2, and NF-κB were obtained from Santa Cruz Biotechnology (Santa Cruz, CA, USA). Specific quantitative PCR (qPCR) primers for ABCA1, PPARγ, LXR-α, LOX-1, CD36, TNF-α, COX-2, mTOR, Foxp3, progranulin (PGRN), and β-actin were purchased from Bioneer (Daejeon, Republic of Korea). Unless stated otherwise, chemical reagents and extraction solvents were acquired from Sigma-Aldrich (St. Louis, MO, USA) or Biosesang (Seongnam, Republic of Korea).

## A. hookeri *root water extract preparation*

To prepare the AHWE, root powder was mixed with distilled water at a 10:1 (v/w) ratio. The mixture was subjected to two rounds of reflux extraction at 95°C for 4 h each. After cooling, the extract was filtered, then concentrated under reduced pressure using a rotary vacuum evaporator (N-1000, EYELA, Tokyo, Japan), and subsequently freeze-dried to determine the solid yield. The final yield of AHWE was calculated to be 34.25%, and the dried extract was stored at –80°C until further use.

### Animals and diet

Four-week-old male C57BL/6 mice were obtained from Orient Bio Co. (Gyeonggi-do, South Korea). Four-week-old mice were used to initiate dietary intervention at a young age and to investigate metabolic and inflammatory responses to 13 weeks of HFCD exposure, consistent with our previous experimental protocol. The animals were acclimated under standardized laboratory conditions, including a temperature range of 20–25°C, relative humidity of 55–60%, and a 12-h light/dark cycle, with unrestricted access to food and water. Prior to dietary intervention, all mice were fed a standard AIN-93G diet (Saeronbio Inc., Gyeonggi-do, South Korea) for 1 week. Following acclimation, the mice were randomly divided into four experimental groups (*n* = 10 per group) and subjected to a 13-week feeding regimen with the following diets: a control group receiving the AIN-93G diet (CON), a group receiving the HFCD (60% kcal from fat plus 1% cholesterol), a group fed the HFCD supplemented with 300 mg/kg/day of AHWE (HFCD + AHWE 300), and another group receiving the HFCD with 500 mg/kg/day of AHWE (HFCD + AHWE 500). Body weight and dietary intake were monitored at 3-day intervals throughout the study. A schematic overview of the in vivo experimental design is presented in Fig. 1. All animal procedures were conducted in accordance with institutional guidelines and approved by the Animal Care and Use Committee of Chonnam National University (CNU IACUC-YB-2023-85).

**Fig. 1:**
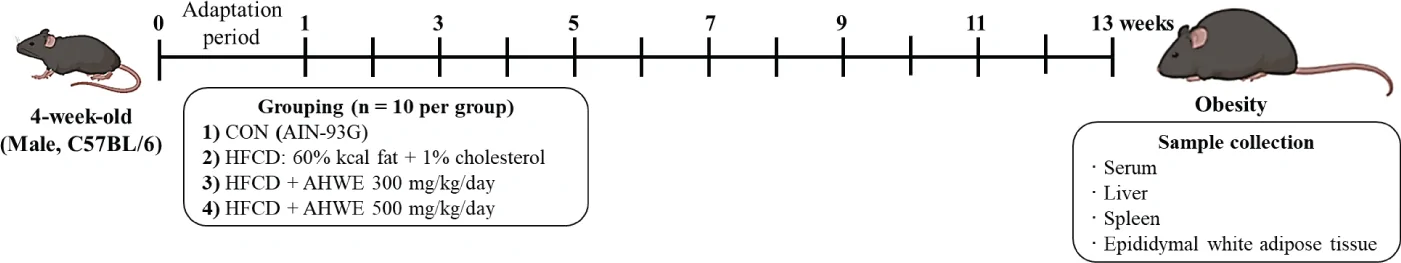
Schematic drawing of the experimental plan for the in vivo study performed in a mouse model. AHWE: *Allium hookeri* water extract; CON: control group (AIN-93G diet containing 7% kcal from fat); HFCD: high-fat and high-cholesterol diet containing 60% kcal from fat and 1% cholesterol.

### Histopathological examination of aorta and liver tissues

Liver and aortic tissues obtained from representative animals in each group were used for histological evaluation. For aortic tissue collection, the thoracic and abdominal cavities were opened after euthanasia, and the internal organs were carefully removed to expose the aorta. The aorta was identified based on its anatomical location along the vertebral column and carefully dissected from the surrounding tissues using fine forceps and scissors. Perivascular adipose and connective tissues surrounding the aorta were carefully removed to minimize contamination while avoiding damage to the aortic wall. The collected liver and aortic tissues were fixed in 10% neutral-buffered formalin (pH 7.0). After fixation, tissues were rinsed with phosphate-buffered saline (PBS), followed by paraffin embedding. The embedded tissues were sectioned into 4–5 µm slices, mounted onto microscope slides, and subsequently stained with hematoxylin and eosin (H&E) for histological evaluation. Each stained section was observed under a light microscope (Leica Microsystems, Wetzlar, Germany) at magnifications of ×100 or ×200. The diameter was analyzed using i-Solution Auto Plus software (IMT i-Solution Inc., Vancouver, BC, Canada). For quantitative histological analysis, hepatocellular vacuolated areas were quantified using ImageJ software (National Institutes of Health, Bethesda, MD, USA). Vacuolated areas were identified using a consistent threshold across all images and expressed as a percentage of the total tissue area within each region of interest (ROI).

### Biochemical analysis

Following blood collection, serum levels of aspartate aminotransferase (AST), alanine aminotransferase (ALT), total cholesterol (TC), triglycerides (TGs), low-density lipoprotein cholesterol (LDL-C), and high-density lipoprotein cholesterol (HDL-C) were measured using commercially available assay kits (Asan Pharmaceutical, Seoul, South Korea). The atherogenic index (AI) was calculated using the established formula: AI = (TC-HDL-C) / HDL-C (30).

### Tissue lysis and protein extraction

Whole-cell lysates were obtained using RIPA Buffer (Biosesang, Sungnam, Korea) supplemented with Halt™ Protease and Phosphatase Inhibitor Cocktail (Thermo Fisher Scientific, Waltham, MA, USA). For nuclear protein extraction, 10% NP-40 was added to a nuclear lysis buffer composed of 20 mM HEPES, 0.4 mM NaCl, 1 mM EDTA, 1 mM EGTA, 1 mM DTT, and 1 mM PMSF. Protein concentrations were quantified using the BCA assay (Pierce, IL, USA) according to the manufacturer’s protocol.

### Histone extraction and purification

Core histones were extracted from liver tissues using the Histone Purification Mini Kit (Active Motif, Carlsbad, CA, USA) according to the manufacturer’s protocol. Briefly, liver samples were homogenized on ice in an extraction buffer using a Dounce homogenizer mounted on a rotating platform. The homogenate was centrifuged at 16,000 × g for 5 min at 4°C, and the pH of the resulting supernatant was adjusted to 8.0 with a neutralization buffer. After equilibrating the spin column with the appropriate buffer, the histone-containing supernatant was applied to the column. The column was washed three times with the wash buffer, and histones were eluted using the elution buffer. Eluted proteins were precipitated overnight with 4% perchloric acid (PCA). The following day, samples were centrifuged at 16,000 × g for 1 h at 4°C and sequentially washed with acetone and PCA. Purified histones were then dissolved in DEPC-treated water, quantified, and stored for further analysis.

### Immunoblotting analysis

Immunoblotting analysis was conducted to determine the expression levels of proteins associated with inflammation, lipid accumulation, cholesterol efflux, and histone acetylation. SDS-PAGE separated protein (20–40 μg) and protein bands were transferred to a nitrocellulose membrane (Invitrogen, Waltham, MA, USA), which was subsequently reacted for 2 h in blocking buffer (10 mM Tris-HCl, pH 7.5; 150 mM NaCl, 0.1% Tween 20, and 5% nonfat dry milk). The membrane was incubated with the following primary antibodies: NF-κB, COX-2, TNF-α, IL-6, SR-A1, CD36, LOX-1, LXR-α, PPARγ, phosphorylated mTOR (p-mTOR), phosphorylated AMPK (p-AMPK), ABCA1, and Foxp3, followed by horseradish peroxidase-conjugated (HRP-conjugated) anti-mouse IgG and anti-rabbit IgG. Afterward, it was washed three times at 10-min intervals with wash buffer (10 mM Tris-HCl, pH 7.5; 150 mM NaCl, 0.1% Tween 20). The TNF-ɑ, COX-2, NF-κB, LXR-α, AMPK, mTOR, p-mTOR, and β-actin were procured from Santa Cruz Biotechnology (Dallas, TX, USA). ABCA1, SR-A1, CD36, and Foxp3 were procured from Abcam (Cambridge, MA, USA). p-AMPK was obtained from Cell Signaling Technology (Beverly, MA, USA). PPARγ and H3 were procured from CUSABIO (Houston, TX, USA). Specific bands on the membrane were visualized with Chemiluminescence Luminol Reagent (Santa Cruz Biotechnology, Dallas, TX, USA) and were analyzed using the ChemiDoc™ XRS+ Imaging System (Bio-Rad, Hercules, CA, USA). Protein expression intensity was normalized to β-actin or H3 and quantified using ImageJ (a free online image analysis software).

### qPCR analysis

qPCR was employed to evaluate the transcriptional expression of genes involved in inflammation, lipid storage, and cholesterol transport. Total RNA was isolated using TRIzol reagent (Thermo Fisher Scientific, Waltham, MA, USA) in accordance with the manufacturer’s instructions. RNA concentration and purity were determined by measuring absorbance at 260 and 280 nm with a NanoDrop 2000 spectrophotometer (Thermo Fisher Scientific, Waltham, MA, USA). cDNA was synthesized from 1 μg of RNA using the Omniscript RT Kit (QIAGEN, Hilden, Germany). Amplification was performed using SYBR Green chemistry on a CFX96 Touch Real-Time PCR Detection System (Bio-Rad, CA, USA). All reactions were conducted in triplicate. Relative mRNA expression was calculated using the 2^–ΔΔCT^ method, with β-actin serving as the internal control. Primer sequences are listed in Table 1.

**Table 1:** Primer sequences

Gene	Primer	Sequence 5′ to 3′
*COX-2*	Forward	TGAGTACCGCAAACGCTTCTC
	Reverse	TGGACGAGGTTTTTCCACCAG
*TNF*-α	Forward	CCCTCACACTCAGATCATCTTCT
	Reverse	GCTACGACGTGGGCTACAG
*IL-6*	Forward	TACCACTTCACAAGTCGGAGGC
	Reverse	CTGCAAGTGCATCATCGTTGTTC
*NF*-κ*B*	Forward	ACCACTGCTCAGGTCCACTGTC
	Reverse	GCTGTCACTATCCCGGAGTTCA
*LOX-1*	Forward	CATCCTCTGCCTGGTGTTGT
	Reverse	TCCTGCTGAGTAAGGTTCGC
*PPAR*γ	Forward	ATTCTGGCCCACCAACTTCGG
	Reverse	TGGAAGCCTGATGCTTTATCCCCA
*LXR*-ɑ	Forward	AGGGATAGGGTTGGAGTC
	Reverse	TTCAAGCGGATCTGTTCT
*ABCA1*	Forward	TGGGAACTCCTGCTAAAAT
	Reverse	CCATGTGGTGTGTAGACA
*mTOR*	Forward	ACCGGCACACATTTGAAGAAG
	Reverse	CTCGTTGAGGATCAGCAAGG
*Foxp3*	Forward	CCTATGGCTCCTTCCTTGGC
	Reverse	ATGAAGTGTGGTCTGTCCTGG
β-*actin*	Forward	AGGCAACGGAACAGGCTATG
	Reverse	AATACCTCCACTTACCTTGGCG

### Enzyme-linked immunosorbent assay

Cell-free supernatants were collected, along with mouse serum collected immediately after sacrifice, and stored at –80°C until assay. Inflammatory factors TNF-α and IL-6 were measured using an ELISA kit (RayBiotech, Norcross, GA, USA). All procedures were performed according to the manufacturer’s protocol.

### Splenocyte preparation

Spleens were surgically excised from 18-week-old C57BL/6 mice housed for 13 weeks under experimental conditions for immediate use in flow cytometry analysis, and from 4-week-old normal C57BL/6 mice for isolation of Treg cells. Splenocytes were collected using a 70 µm cell strainer (SPL) and centrifuged at 1,500 rpm for 3 min at 4°C. Erythrocytes were lysed using 1× Red Blood Cell Lysis Buffer (Sigma-Aldrich), and splenocytes were finally washed in Hanks’ Balanced Salt solution (HBSS, Sigma-Aldrich). To perform the flow cytometry experiments, freshly isolated cells (1 × 10^6^/mL) were pooled, pelleted, and resuspended in 1 mL of PBS. To perform the Tregs isolation, freshly isolated cells (1 × 10^8^/mL) were pooled, pelleted, and resuspended with 1~2 mL of EasySep™ Buffer (STEMCELL Technologies, Vancouver, Canada) and used according to the manufacturer’s instructions. Counting was performed using a TC20 Automated Cell Counter (Bio-Rad).

### Treg cell culture and AHWE treatment

Splenocytes were resuspended in EasySep™ Buffer (STEMCELL Technologies, Vancouver, Canada). CD4^+^CD25^+^ Treg cells were isolated using the EasySep™ Mouse CD4^+^CD25^+^ Regulatory T Cell Isolation Kit II (STEMCELL Technologies, Cat#18783) following the manufacturer’s instructions. Briefly, splenocytes were first incubated with CD4^+^ T Cell Isolation Cocktail and Streptavidin RapidSpheres™, and negatively selected using an EasySep™ magnet to enrich for CD4^+^ T cells. The pre-enriched cells were subsequently incubated with Mouse FcR Blocker, CD25 Positive Selection Cocktail, PE Selection Cocktail, and Dextran RapidSpheres™ to isolate CD25^+^ cells. Magnetic separation was repeated four times to increase purity. Then, isolated Tregs were cultured in a 6-well plate (1 × 10^6^ cells/well) for 24 h with Roswell Park Memorial Institute (RPMI) 1640 medium (Welgene, Daegu, South Korea) supplemented with 10% fetal bovine serum (FBS), 1% antibiotics (Welgene), 10 mM HEPES, 1 mM sodium pyruvate, and 50 µM β-mercaptoethanol (Sigma-Aldrich). The culture was maintained in an atmosphere of 5% CO_2_ at 37°C. To maintain Treg survival and function, IL-2 (2,000 IU/mL; Sigma-Aldrich) was supplemented in the culture medium in 6-well plates. AHWE (dissolved in RPMI medium) was used for cell treatment. Tregs were cultured in the absence or presence of various concentrations of AHWE (31–250 μg/mL) for 48 h. After 48 h of incubation, the medium was saved for the measurement of IL-10, and cells were harvested for further analysis, including flow cytometry analysis.

### Flow cytometry

Flow cytometry analysis was conducted to assess the impact of AHWE on Treg populations in both HFCD-fed mice and isolated Tregs. Dead cells were excluded using the Live/Dead Fixable Aqua Dead Cell Stain Kit (Invitrogen, USA) according to the manufacturer’s guidelines. The following fluorochrome-conjugated monoclonal antibodies against mouse antigens were used: CD4-FITC, CD25-PE, and Foxp3-APC (all from R&D Systems [R&D], Minneapolis, MN, USA). Cells were incubated with 10 μL of each antibody at 4°C for 30 min. For intracellular detection of Foxp3, cells were washed twice with flow staining buffer and fixed using fixation buffer Becton, Dickinson and Company (BD Biosciences). After 30 min incubation at 4°C, cells were again washed with Perm/Wash Buffer (BD Biosciences) and stained for intracellular Foxp3 expression at room temperature for 30 min following the manufacturer’s protocol. Subsequently, cells were washed twice with staining buffer and resuspended in 400 μL of buffer. Data acquisition was performed using a CytoFLEX flow cytometer (Beckman Coulter, CA, USA).

### Statistical analysis

All experiments were performed at least three times. Multiple comparisons were performed using one-way analysis of variance (ANOVA), followed by Duncan’s multiple range test using SPSS version 25.0 (SPSS Institute, Chicago, IL, USA). The specific significance values are provided in the figure legends, and statistical significance was defined as *P* < 0.05.

## Results

### AHWE supplementation reduced body weight, organ weights, and food efficiency in HFCD-fed mice

In this study, mice were considered obese if their body weight was at least 20% higher than that of age-matched normal diet-fed controls, a criterion commonly used in rodent models of diet-induced obesity (31). As presented in Table 2, body weight was markedly increased in the HFCD group following 13 weeks of HFCD feeding, relative to the CON group (*P* < 0.05). In contrast, AHWE supplementation led to significantly lower body weight gain relative to the HFCD group (*P* < 0.05). Importantly, food consumption did not differ significantly among the groups. The food efficiency ratio (FER) was lower in the CON group than in the three groups fed an HFCD (*P* < 0.05). Furthermore, the HFCD + AHWE 500 group showed a reduced FER compared to the HFCD group (*P* < 0.05). Liver tissue weights were elevated in the HFCD group compared to the CON group (*P* < 0.05); however, those in the HFCD + AHWE 500 group were significantly reduced relative to the HFCD (*P* < 0.05). Similarly, the weights of epididymal and retroperitoneal adipose tissues were increased in the HFCD group relative to the CON group (*P* < 0.05). Although a reduction was observed in the HFCD + AHWE 300 group compared to the HFCD group, the change was not statistically significant. In contrast, the HFCD + AHWE 500 group exhibited significantly lower epididymal and retroperitoneal adipose tissue weights than the HFCD group (*P* < 0.05). Given that obesity is a major risk factor for the development of atherosclerosis, these findings suggest that AHWE may attenuate diet-induced obesity by reducing body weight gain and improving FER, thereby potentially contributing to the prevention of atherosclerosis.

**Table 2:** Effects of AHWE on the body, organ weights and food efficiency in HFCD-fed mice

Parameters	CON (*n* = 10)	HFCD (*n* = 10)	HFCD + AHWE 300 (*n* = 10)	HFCD + AHWE 500 (*n* = 10)
Baseline body weight (g)	19.34 ± 0.65	19.45 ± 1.03	19.01 ± 0.87	19.35 ± 0.87
Final body weight (g)^1)^	32.74 ± 1.04ᶜ	46.20 ± 1.47ᵃ	44.25 ± 1.61ᵃ	39.52 ± 1.18ᵇ
Body weight gain (g)	13.7 ± 0.69ᶜ	26.98 ± 1.05ᵃ	25.68 ± 1.06ᵃ	20.01 ± 0.83ᵇ
Average daily food intake (g/day)	2.86 ± 0.10	3.03 ± 0.10	3.03 ± 0.11	3.26 ± 0.13
Food efficiency (%)^2)^	5.15 ± 0.26ᶜ	9.57 ± 0.37ᵃ	9.11 ± 0.38ᵃ	6.60 ± 0.27ᵇ
Liver (g)	1.07 ± 0.05ᶜ	2.71 ± 0.25ᵃ	2.38 ± 0.27ᵃᵇ	1.83 ± 0.14ᵇ
Epididymal white adipose tissue (g)	1.21 ± 0.15ᵇ	2.37 ± 0.14ᵃ	2.26 ± 0.16ᵃ	2.24 ± 0.09ᵃ
Retroperitoneal adipose tissue (g)	0.49 ± 0.06ᶜ	1.08 ± 0.07ᵃ	1.03 ± 0.09ᵃ	0.63 ± 0.02ᵇ

Body weight was measured once every 3 days throughout the experimental period. 1) Body weights after 13 weeks in C57BL/6 mice. 2) Food efficiency ratio was calculated as follows: (body weight gain (g)/total food intake (g)) × 100. AHWE: *Allium hookeri* water extract; CON: control group (AIN-93G diet containing 7% kcal from fat); HFCD: high-fat and high-cholesterol diet containing 60% kcal from fat and 1% cholesterol; HFCD + AHWE 300: HFCD supplemented with 300 mg/kg/day AHWE; HFCD + AHWE 500: HFCD supplemented with 500 mg/kg/day AHWE. Data are expressed as mean ± SEM (*n* = 10). Different letters indicate significant differences (*P* < 0.05) as determined by Duncan’s multiple range test.

### AHWE supplementation improved metabolic parameters in HFCD-fed mice

As shown in Table 3, both the AST and ALT levels were increased in the HFCD group compared to the CON group (*P* < 0.05) but decreased in the HFCD + AHWE 500 group compared to the HFCD group (*P* < 0.05), suggesting a hepatoprotective effect of AHWE. Dyslipidemia, a key contributor to atherosclerosis and CVD, was also assessed. The HFCD increased the TG levels compared to the CON diet (*P* < 0.05). AHWE supplementation reduced the TG levels compared to the HFCD (*P* < 0.05). Similarly, the TC and LDL-C levels were significantly higher in the HFCD group than in the CON group (*P* < 0.01), whereas AHWE supplementation decreased both parameters (*P* < 0.05). Conversely, the HDL-C levels were reduced by HFCD feeding (*P* < 0.05) but were significantly restored in AHWE-supplemented mice (*P* < 0.05). The AI, which reflects the risk of cholesterol-related vascular damage, was increased in the HFCD group relative to the CON group (*P* < 0.05). AHWE supplementation lowered the AI values relative to the HFCD (*P* < 0.05), indicating a potential to reduce atherosclerotic risk. In addition, hyperinsulinemia – a hallmark of insulin resistance that promotes vascular inflammation – was observed in the HFCD group, with higher insulin levels compared to the CON group (*P* < 0.05). These elevated levels were decreased in the HFCD + AHWE 500 group (*P* < 0.05). Fasting blood glucose levels followed a similar trend, with increased values in the HFCD group (*P* < 0.01), which were reduced by AHWE supplementation (*P* < 0.05). This suggests a beneficial role of AHWE in improving insulin sensitivity.

**Table 3:** Effects of AHWE on plasma metabolic parameter in HFCD-fed mice

Parameters	CON (*n* = 10)	HFCD (*n* = 10)	HFCD + AHWE 300 (*n* = 10)	HFCD + AHWE 500 (*n* = 10)
AST (U/L)	58.67 ± 9.54^c^	135.75 ± 10.43^a^	110.33 ± 17.46^ab^	69.29 ± 13.11^bc^
ALT (U/L)	43.26 ± 13.77^b^	95.31 ± 10.66^a^	80.04 ± 7.71^a^	50.22 ± 6.40^b^
TC (mg/dL)	77.32 ± 4.72^c^	193.27 ± 13.05^a^	145.27 ± 9.11^b^	100.98 ± 8.99^c^
TG (mg/dL)	76.96 ± 4.01^c^	117.40 ± 4.01^a^	92.83 ± 1.54^b^	78.10 ± 4.91^c^
HDL-C (mg/dL)	71.97 ± 5.11^a^	33.75 ± 1.77^b^	61.14 ± 4.17^a^	73.31 ± 4.33^a^
LDL-C (mg/dL)	32.37 ± 5.35^c^	156.29 ± 13.41^a^	102.25 ± 10.18^b^	56.04 ± 8.85^c^
Serum insulin (ng/mL)	1.11 ± 0.12^b^	4.05 ± 0.64^a^	2.18 ± 0.33^b^	1.23 ± 0.21^b^
Fasting glucose (mg/dL)	115.20 ± 3.53^c^	248.30 ± 8.00^a^	199.67 ± 10.22^ab^	196.50 ± 5.50^b^
AI	0.42 ± 0.07^b^	4.89 ± 0.57^a^	1.51 ± 0.27^b^	0.56 ± 0.15^b^

AST: aspartate aminotransferase; ALT: alanine aminotransferase; TC: total cholesterol; TG: triglycerides; HDL-C: high-density lipoprotein cholesterol; LDL-C: low-density lipoprotein cholesterol; AI: atherogenic index, calculated as (TC – HDL-C)/HDL-C; AHWE: *Allium hookeri* water extract; CON: control group (AIN-93G diet containing 7% kcal from fat); HFCD: high-fat and high-cholesterol diet containing 60% kcal from fat and 1% cholesterol; HFCD + AHWE 300: HFCD supplemented with 300 mg/kg/day AHWE; HFCD + AHWE 500: HFCD supplemented with 500 mg/kg/day AHWE. Data are expressed as mean ± SEM (*n* = 10). Different letters indicate significant differences (*P* < 0.05) as determined by Duncan’s multiple range test.

### AHWE supplementation ameliorated hepatic injury in HFCD-fed mice

Representative gross images of the liver and epididymal adipose tissue are shown in Fig. 2A. To evaluate the protective effect of AHWE on hepatic lipid accumulation in HFCD-fed mice, we observed lipid vacuoles using H&E-stained hepatic sections, as shown in Fig. 2B. Compared to the CON group, marked hepatic vacuolation was observed in the HFCD group, whereas AHWE supplementation attenuated these histological changes. Specifically, the HFCD group exhibited numerous clear cytoplasmic vacuoles consistent with hepatic lipid accumulation, whereas AHWE treatment reduced the extent of these vacuolar changes. Quantitative analysis of the H&E-stained liver sections showed that the hepatic vacuolated area was significantly increased in the HFCD group compared with the CON group (*P* < 0.05), whereas AHWE supplementation significantly reduced the vacuolated area compared with the HFCD group (*P* < 0.05) (Fig. 2C). Consistent with the liver weight presented in Table 1, these findings suggest that AHWE supplementation may alleviate hepatic lipid accumulation by reducing lipid accumulation in the liver of HFCD-fed mice. Given that metabolic dysfunction-associated steatotic liver disease (MASLD) is closely associated with the development and progression of atherosclerosis, AHWE may have potential anti-atherosclerotic effects.

**Fig. 2:**
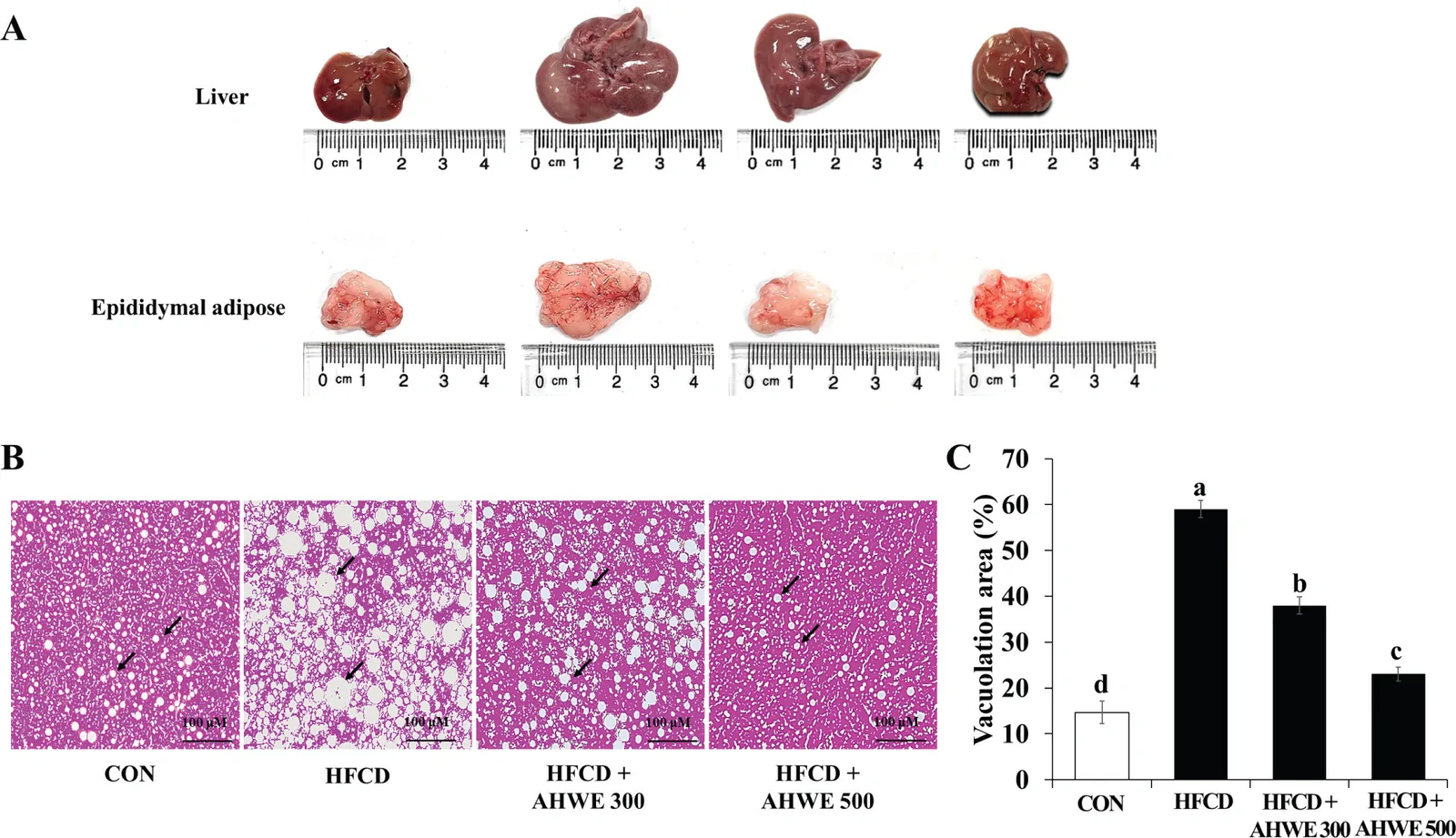
Effects of AHWE on lipid accumulation in liver and epididymal adipose tissue of HFCD-fed mice. (A) Representative images of the liver and epididymal white adipose tissue of male C57BL/6 mice from each group at the end of the experiment. After sacrifice, liver and epididymal white adipose tissues were excised and photographed to assess gross morphological changes. (B) Histological evaluation of liver tissue in HFCD-fed mice treated with AHWE. Representative photomicrographs of hematoxylin and eosin (H&E)-stained liver sections show intracellular hepatocellular vacuolation (white areas) in hepatocytes. Arrows indicate clear cytoplasmic vacuoles associated with hepatic lipid accumulation. Images were obtained from formalin-fixed, paraffin-embedded liver sections at ×100 magnification (scale bars = 100 μm). (C) Quantification of hepatic vacuolated area in H&E-stained liver sections, expressed as a percentage of the total tissue area. AHWE: *Allium hookeri* water extract; CON: control group (AIN-93G diet containing 7% kcal from fat); HFCD: high-fat and high-cholesterol diet containing 60% kcal from fat and 1% cholesterol; HFCD + AHWE 300: HFCD supplemented with 300 mg/kg/day AHWE; HFCD + AHWE 500: HFCD supplemented with 500 mg/kg/day AHWE; ANOVA: analysis of variance. Data are expressed as mean ± SEM (*n* = 10). Different letters indicate significant differences among groups (*P* < 0.05), as determined by one-way ANOVA followed by Duncan’s multiple range test.

### AHWE supplementation ameliorated aorta injury in HFCD-fed mice

To evaluate the histological characteristics of the aorta, sections were stained with H&E, as shown in Fig. 3. Structurally, the arterial wall consists of three anatomically defined layers: the intima (the inner endothelial lining), the media (a central layer rich in smooth muscle), and the adventitia (the outermost layer composed of connective tissue). Atherosclerotic lesions typically begin with endothelial dysfunction in the intima, which increases vascular permeability and facilitates the accumulation of lipids and immune cells between the intimal and medial layers, leading to intimal thickening and luminal narrowing. In the CON group, no pathological changes were observed in the aortic structure. In contrast, HFCD-fed mice revealed marked intimal thickening. However, the AHWE-treated group showed less pronounced intimal thickening than the HFCD group. Moreover, the mean aortic wall thickness was increased in the HFCD group relative to the CON group (*P* < 0.05). AHWE treatment attenuated this thickening, resulting in a marked decrease in wall thickness relative to the HFCD group (*P* < 0.05). These observations suggest that AHWE may exert a potential protective effect against atherosclerotic lesion development.

**Fig. 3:**
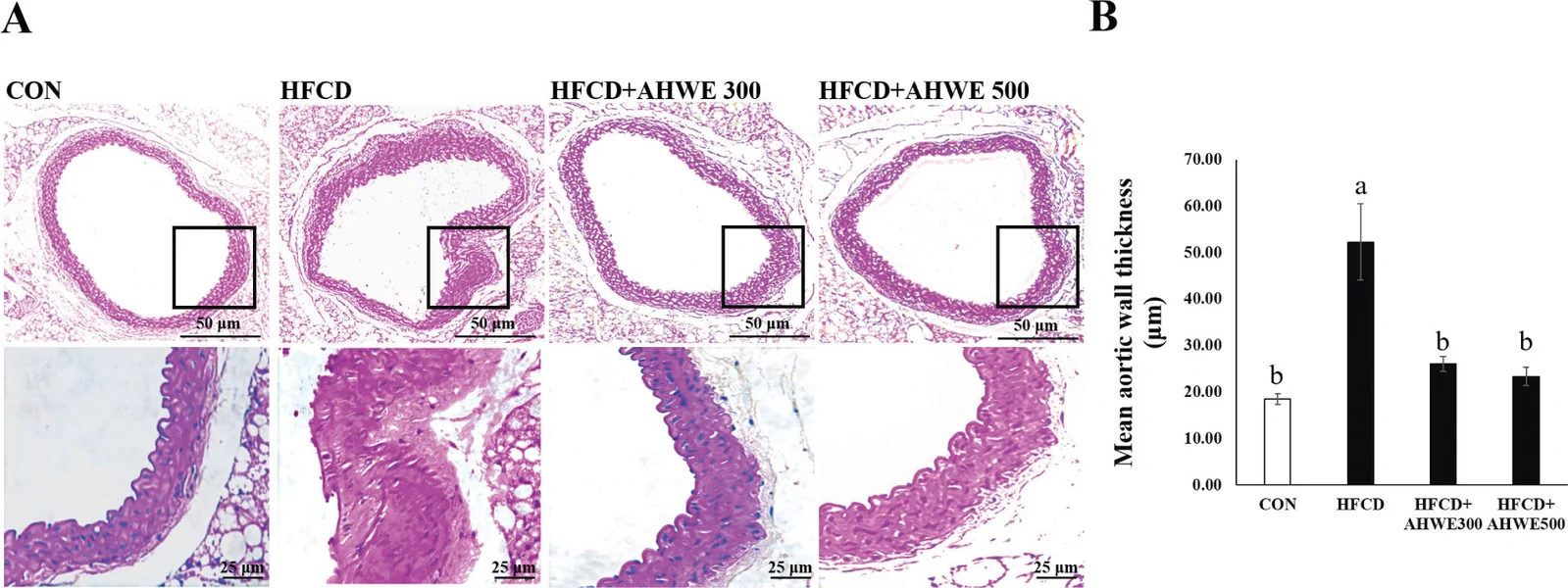
Effect of AHWE on the aorta of HFCD-fed mice. (A) Representative histological images of aortic tissue stained with hematoxylin and eosin (H&E). Sections were prepared from formalin-fixed, paraffin-embedded samples and captured at ×40 (scale bar = 50 μm) and ×200 (scale bar = 25 μm) magnifications. The upper and lower panels represent low- and high-magnification images, respectively, from the same experimental group. (B) Quantification of mean aortic wall thickness. The thickness of the aortic wall was measured using ImageJ software. Multiple measurements were taken from each section and averaged to determine the mean thickness for each mouse. AHWE: *Allium hookeri* water extract; CON: control group (AIN-93G diet containing 7% kcal from fat); HFCD: high-fat and high-cholesterol diet containing 60% kcal from fat and 1% cholesterol; HFCD + AHWE 300: HFCD supplemented with 300 mg/kg/day AHWE; HFCD + AHWE 500: HFCD supplemented with 500 mg/kg/day AHWE. Data are expressed as mean ± SEM (*n* = 10). Different letters indicate significant differences (*P* < 0.05) as determined by one-way ANOVA followed by Duncan’s multiple range test.

### AHWE supplementation upregulated the expression of genes involved in the reverse cholesterol transport pathway in the liver of HFCD-fed mice

The reverse cholesterol transport pathway, mediated by the PPARγ–LXR-α–ABCA1 signaling cascade, is a critical process that maintains cholesterol homeostasis by promoting the efflux of excess cholesterol from peripheral tissues, including the arterial wall, to the liver for excretion (32). The expression levels of the PPARγ, LXR-α, and ABCA1 genes, which are involved in cholesterol efflux, were evaluated using immunoblotting and qPCR. For immunoblotting, PPARγ and LXR-α were analyzed using liver nuclear extracts, whereas ABCA1 was analyzed using whole-liver lysates. As illustrated in Fig. 4A–D, although the protein levels of LXR-α and ABCA1 were significantly decreased in the HFCD group (*P* < 0.05). Consistently, as shown in Fig. 4E–G, the mRNA expression levels of PPARγ, LXR-α, and ABCA1 were also decreased in the HFCD group relative to the CON group (*P* < 0.05). Notably, AHWE supplementation upregulated the expression of PPARγ, LXR-α, and ABCA1 relative to the HFCD group (*P* < 0.05). These findings suggest that AHWE may exert protective effects against atherosclerosis by upregulating cholesterol efflux-associated expression of genes such as PPARγ, LXR-α, and ABCA1. Considering the critical role of lipid homeostasis in the development and progression of atherosclerosis, these results indicate that AHWE supplementation may contribute to the maintenance of cholesterol homeostasis and attenuate atherogenic risk in HFCD-fed mice.

**Fig. 4:**
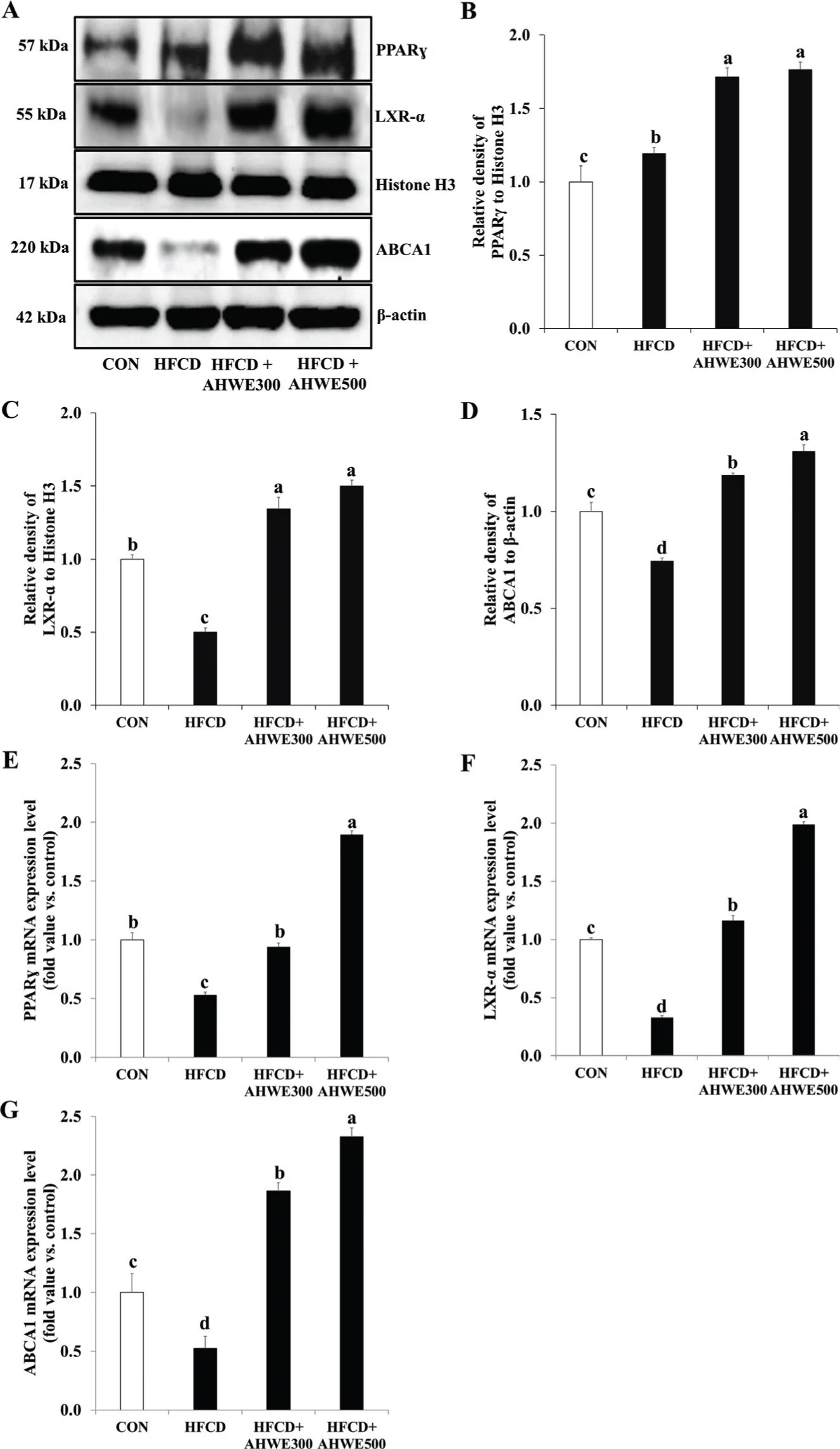
Effect of AHWE on the reverse cholesterol transport pathway in the liver of HFCD-fed mice. (A) Protein expression levels of ABCA1, LXR-α, and PPARγ in liver tissue were assessed by immunoblotting using whole-liver lysates. (B-D) Equal loading of proteins was confirmed by reprobing for β-actin and histone H3 following membrane stripping. (E–G) Hepatic mRNA expression levels of ABCA1, LXR-α, and PPARγ were quantified using qPCR and analyzed by the 2^–ΔΔCT^ method. Expression levels were normalized to β-actin. AHWE: *Allium hookeri* water extract; CON: control group (AIN-93G diet containing 7% kcal from fat); HFCD: high-fat and high-cholesterol diet containing 60% kcal from fat and 1% cholesterol; HFCD + AHWE 300: HFCD supplemented with 300 mg/kg/day AHWE; HFCD + AHWE 500: HFCD supplemented with 500 mg/kg/day AHWE; ABCA1: ATP-binding cassette subfamily A member 1; LXR-α: liver X receptor alpha; PPARγ: peroxisome proliferator-activated receptor gamma; qPCR: quantitative polymerase chain reaction. Data are expressed as mean ± SEM (*n* = 5). Different letters indicate significant differences (*P* < 0.05) as determined by one-way ANOVA followed by Duncan’s multiple range test.

### AHWE supplementation downregulated the expression of genes involved in lipid uptake in the liver of HFCD-fed mice

In chronic inflammatory conditions, such as obesity and atherosclerosis, the upregulation of scavenger receptors responsible for the uptake of modified cholesterol (CD36, SR-A1, and LOX-1) disrupts hepatic lipid homeostasis by promoting lipid accumulation, thereby contributing to the progression of atherosclerosis. To assess the effect of AHWE on the expression of CD36, SR-A1, and LOX-1 under atherosclerotic conditions, immunoblotting was performed using whole-liver lysates. As shown in Fig. 5A–D, the expression levels of CD36, SR-A1, and LOX-1 were increased in the HFCD group compared to the CON group (*P* < 0.05). AHWE supplementation reduced the expression of these scavenging receptors (*P* < 0.05). Additionally, the mRNA expression of LOX-1 was determined using qPCR. Figure 5E demonstrates that LOX-1 mRNA levels were upregulated in the HFCD group relative to the CON group, but significantly downregulated following AHWE supplementation (*P* < 0.05). These observations indicate a potential atherosclerosis protective role of AHWE by alleviating lipid accumulation in the liver through the downregulation of scavenger receptors such as CD36, SR-A1, and LOX-1.

**Fig. 5:**
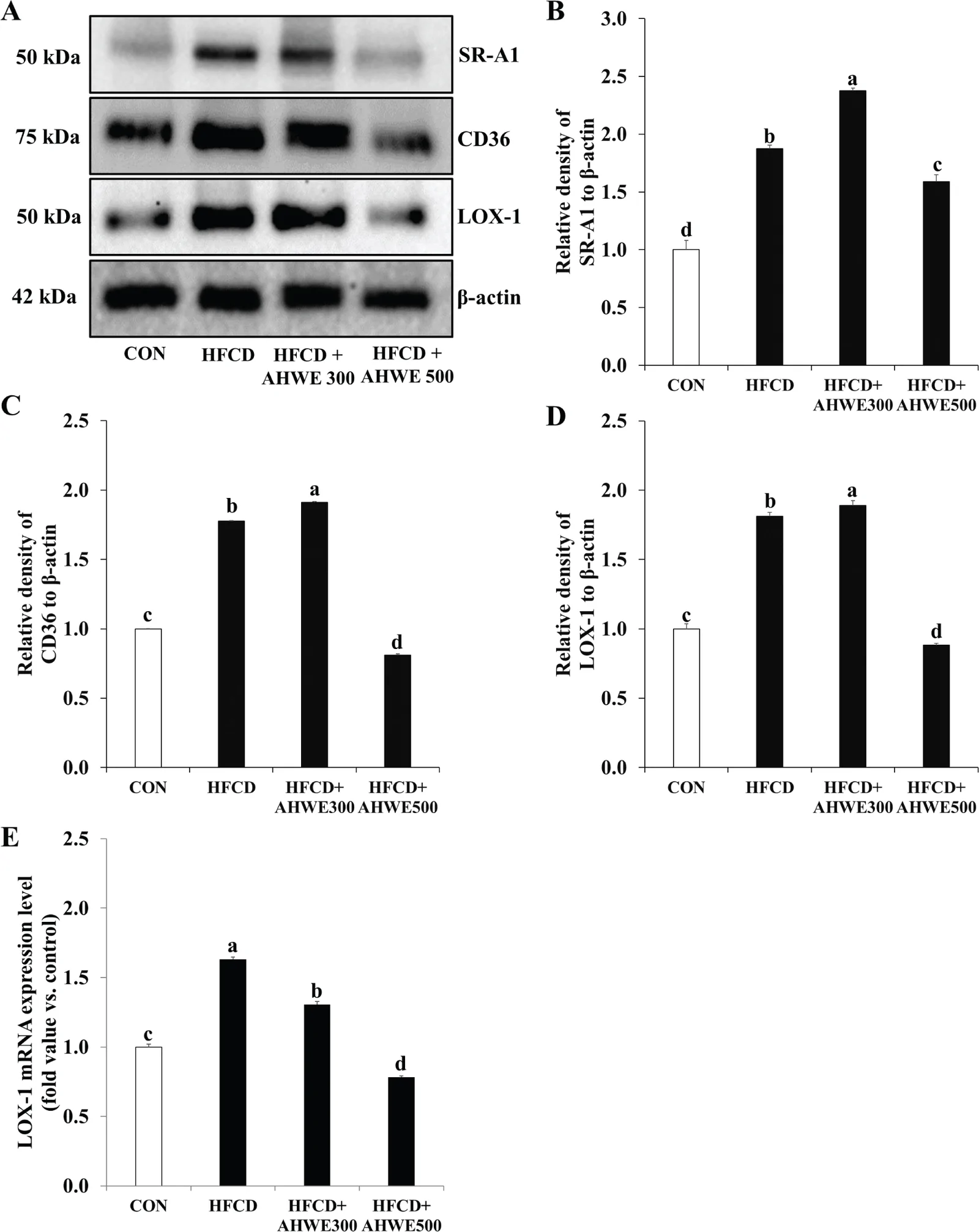
Effect of AHWE on lipid accumulation in the liver of HFCD-fed mice. (A) Protein expression levels of SR-A1, CD36, and LOX-1 in liver tissue were assessed by immunoblotting using whole-liver lysates. (B–D) Equal loading of proteins was confirmed by reprobing for β-actin following membrane stripping. (E) Hepatic mRNA expression levels of LOX-1 were quantified using qPCR and analyzed by the 2^–ΔΔCT^ method. Expression levels were normalized to β-actin. AHWE: *Allium hookeri* water extract; CON: control group (AIN-93G diet containing 7% kcal from fat); HFCD: high-fat and high-cholesterol diet containing 60% kcal from fat and 1% cholesterol; HFCD + AHWE 300: HFCD supplemented with 300 mg/kg/day AHWE; HFCD + AHWE 500: HFCD supplemented with 500 mg/kg/day AHWE; SR-A1: scavenger receptor A1; CD36: cluster of differentiation 36; LOX-1: lectin-like oxidized low-density lipoprotein receptor-1; qPCR: quantitative polymerase chain reaction. Data are expressed as mean ± SEM (*n* = 5). Different letters indicate significant differences (*P* < 0.05) as determined by one-way ANOVA followed by Duncan’s multiple range test.

### AHWE supplementation modulated the expression of genes involved in the AMPK/mTOR pathway in the liver of HFCD-fed mice

Dysregulation of AMPK and mTOR signaling is closely associated with metabolic disorders, including obesity, hepatic steatosis, and atherosclerosis. The protein expression of AMPK and mTOR was analyzed using immunoblotting and whole-liver lysates. As shown in Fig. 6A–C, p-AMPK, which represents its activated state, was significantly downregulated in the HFCD group compared to the CON group (*P* < 0.05). In contrast, AHWE supplementation restored p-AMPK expression, showing a marked increase compared with the HFCD group (*P* < 0.05). Conversely, the expression of p-mTOR, the active form of mTOR, was elevated by the HFCD when compared to the CON diet (*P* < 0.05). These levels were suppressed in the AHWE supplementation group. Furthermore, the mRNA expression of mTOR was determined using qPCR. As illustrated in Fig. 6D, the mRNA expression level of p-mTOR was downregulated in the AHWE supplementation group relative to the HFCD group (*P* < 0.05). AHWE supplementation appears to improve lipid metabolism by modulating the interplay between AMPK signaling and the mTOR pathway, thereby at least partially alleviating atherosclerosis.

**Fig. 6:**
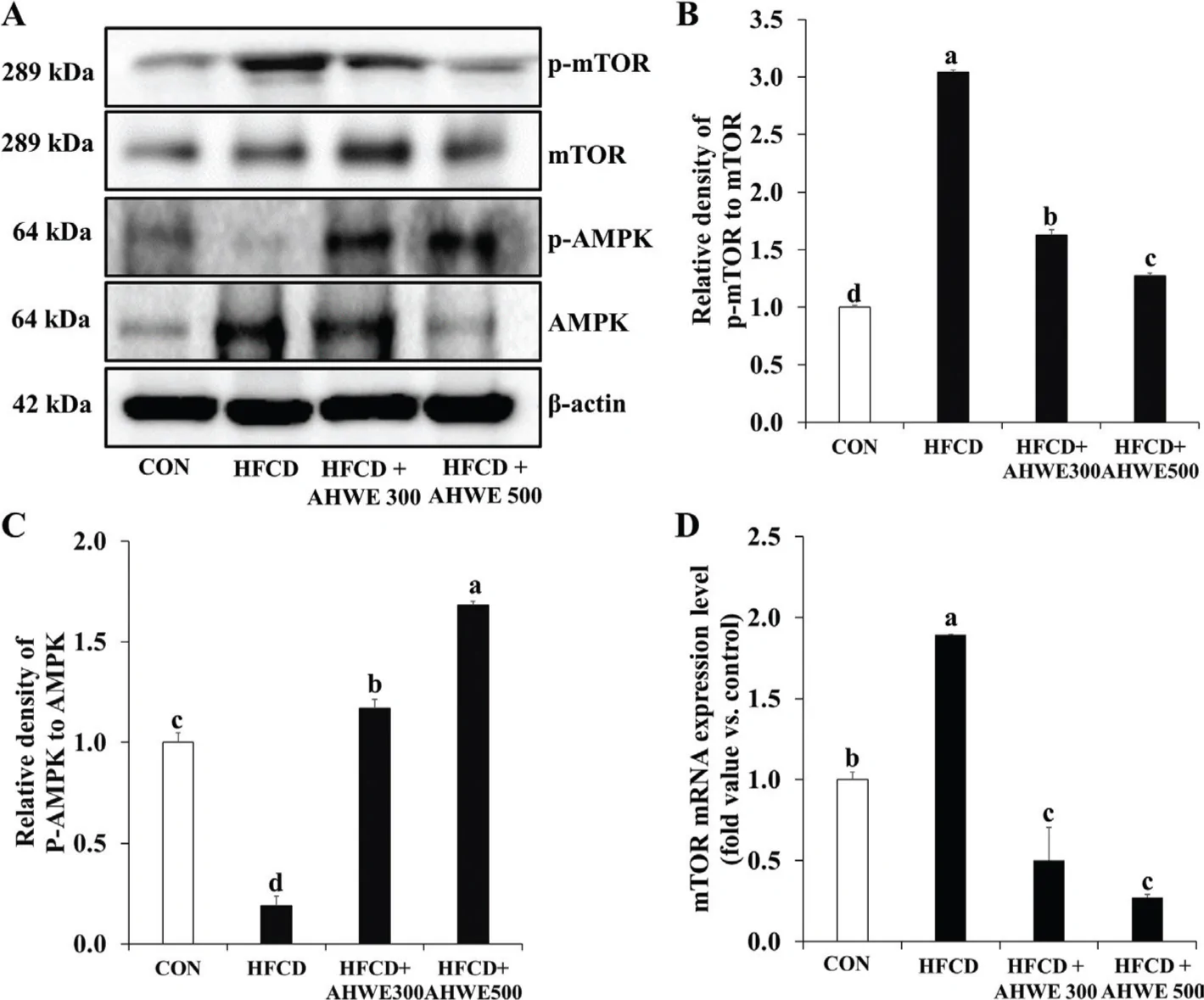
Effect of AHWE on the AMPK/mTOR pathway in the liver of HFCD-fed mice. (A) Protein levels of p-AMPK and p-mTOR were measured by immunoblotting using whole-liver lysates. (B–C) Equal protein loading was confirmed by stripping and reprobing the membranes for β-actin. (D Hepatic mRNA expression of mTOR was analyzed using qPCR and quantified by the 2^–ΔΔCT^ method. Expression levels were normalized to β-actin. AHWE: *Allium hookeri* water extract; CON: control group (AIN-93G diet containing 7% kcal from fat); HFCD: high-fat and high-cholesterol diet containing 60% kcal from fat and 1% cholesterol; HFCD + AHWE 300: HFCD supplemented with 300 mg/kg/day AHWE; HFCD + AHWE 500: HFCD supplemented with 500 mg/kg/day AHWE; p-AMPK: Phosphorylated AMP-activated protein kinase; p-mTOR: Phosphorylated mechanistic target of rapamycin; qPCR: quantitative polymerase chain reaction. Data are expressed as mean ± SEM (*n* = 5). Different letters indicate significant differences (*P* < 0.05) as determined by one-way ANOVA followed by Duncan’s multiple range test.

### AHWE supplementation modulated the expression of pro-inflammatory markers in the liver of HFCD-fed mice

Pro-inflammatory cytokines are significantly upregulated during the progression of atherosclerotic lesions, contributing to sustained inflammatory response. To evaluate the inflammatory responses, the expression levels of COX-2, TNF-α, IL-6, and NF-κB were analyzed by immunoblotting and qPCR. For immunoblotting, COX-2, TNF-α, and IL-6 were analyzed using whole-liver lysates, whereas NF-κB was analyzed using liver nuclear extracts. Figure 7A–E demonstrates that the expression levels of these pro-inflammatory genes were markedly increased in the HFCD group relative to the CON group (*P* < 0.01). In contrast, supplementation with AHWE significantly attenuated their expression relative to the HFCD group (*P* < 0.01). These findings suggest that AHWE supplementation may exert anti-inflammatory effects in HFCD-fed mice by downregulating pro-inflammatory cytokines. Moreover, as shown in Fig. 7F–H, the mRNA expression of COX-2, TNF-α, and NF-κB was significantly upregulated in the HFCD group compared to the CON group (*P* < 0.01). AHWE supplementation led to downregulation of these inflammatory genes relative to the HFCD group (*P* < 0.01). Additionally, the ELISA results (Fig. 7I and 7J) demonstrated that the secretion levels of TNF-α and IL-6 were increased in the HFCD group (*P* < 0.01), but were decreased in the HFCD + AHWE 500 group relative to the HFCD group (*P* < 0.01). These findings suggest that AHWE may contribute to the attenuation of atherosclerosis in HFCD-fed mice through the downregulation of pro-inflammatory cytokines.

**Fig. 7:**
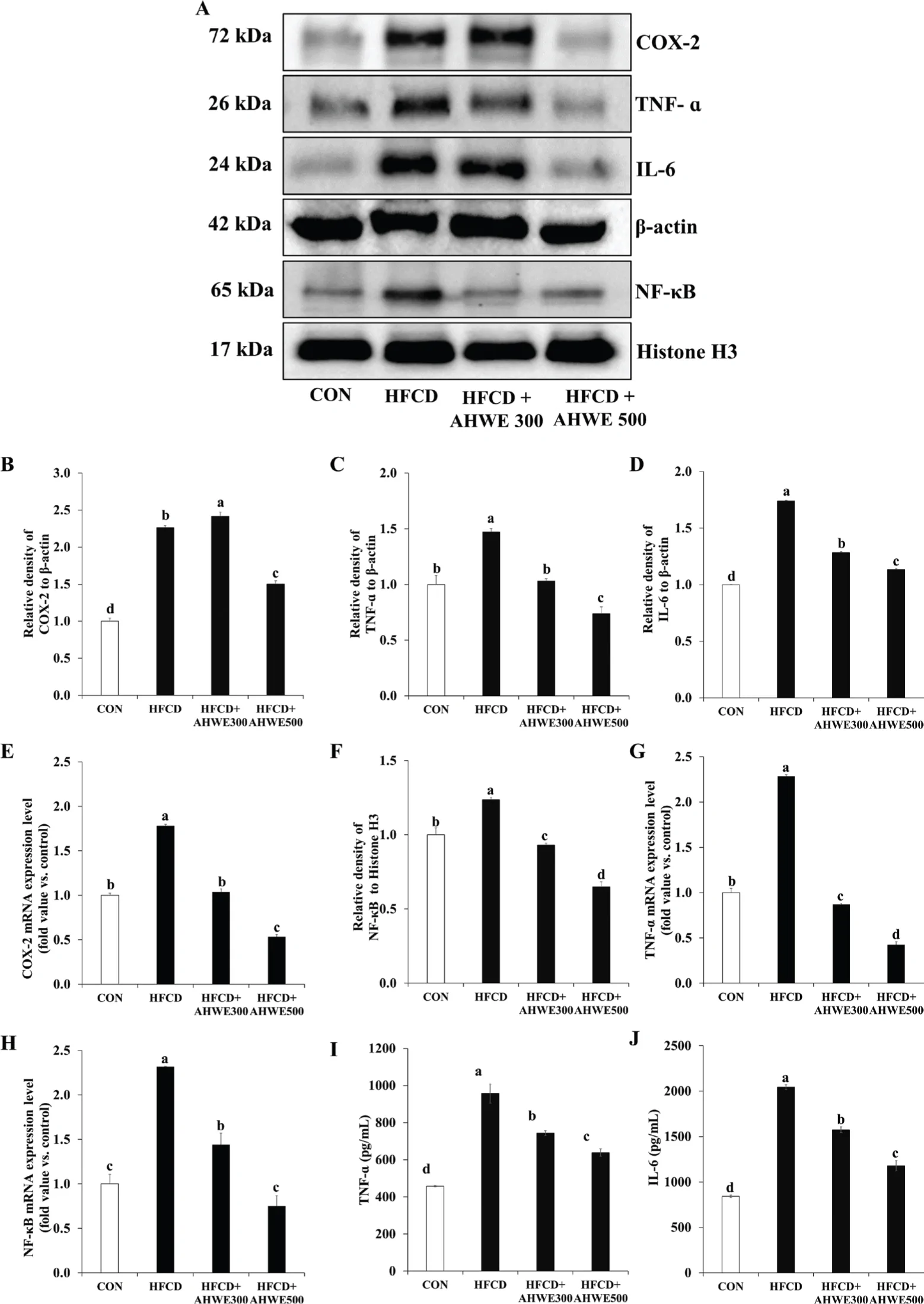
Effect of AHWE on inflammation-related genes in the liver of HFCD-fed mice. (A) Protein levels of TNF-α, COX-2, IL-6, and NF-κB were measured by immunoblotting using whole-liver lysates. (B–F) Equal protein loading was confirmed by stripping and reprobing the membranes for β-actin and histone H3. (G–H) Hepatic mRNA expression levels of TNF-α, COX-2, and NF-κB were quantified using qPCR and analyzed by the 2^–ΔΔCT^ method. Expression levels were normalized to β-actin. (I–J) The secretion levels of TNF-α and IL-6 in serum were measured using ELISA kit. AHWE: *Allium hookeri* water extract; CON: control group (AIN-93G diet containing 7% kcal from fat); HFCD: high-fat and high-cholesterol diet containing 60% kcal from fat and 1% cholesterol; HFCD + AHWE 300: HFCD supplemented with 300 mg/kg/day AHWE; HFCD + AHWE 500: HFCD supplemented with 500 mg/kg/day AHWE; TNF-α: Tumor necrosis factor-alpha; COX-2: Cyclooxygenase-2; IL-6: Interleukin-6; NF-κB: Nuclear factor-kappa B; qPCR: quantitative polymerase chain reaction. Data are expressed as mean ± SEM (*n* = 5). Different letters indicate significant differences (*P* < 0.05) as determined one-way ANOVA followed by Duncan’s multiple range test.

### AHWE supplementation modulated H3K9ac and H3K27ac in the liver of HFCD-fed mice

Histone acetylation, a type of epigenetic modification, plays a significant role in atherosclerosis by regulating gene expression involved in inflammation and plaque formation within the blood vessel wall. Specifically, H3K9ac and H3K27ac have been shown to upregulate the expression of pro-inflammatory cytokines, such as IL-6 and matrix metalloproteinases (MMPs), including MMP-1, -3, -10, -12, and -13, which are critical mediators of plaque development and progression. To confirm whether histone acetylation regulates gene expression during atherosclerosis, H3K9ac and H3K27ac were evaluated in the presence or absence of AHWE supplementation. Histones were isolated from mouse liver tissues to assess H3K9ac and H3K27ac. As illustrated in Fig. 8, both H3K9ac and H3K27ac were elevated in the HFCD group, but significantly reduced in the groups receiving AHWE supplementation compared to the HFCD group (*P* < 0.05). These findings suggest that AHWE suppresses inflammation by decreasing HFCD-induced histone acetylation, thereby moderating pro-inflammatory cytokine transcription.

**Fig. 8:**
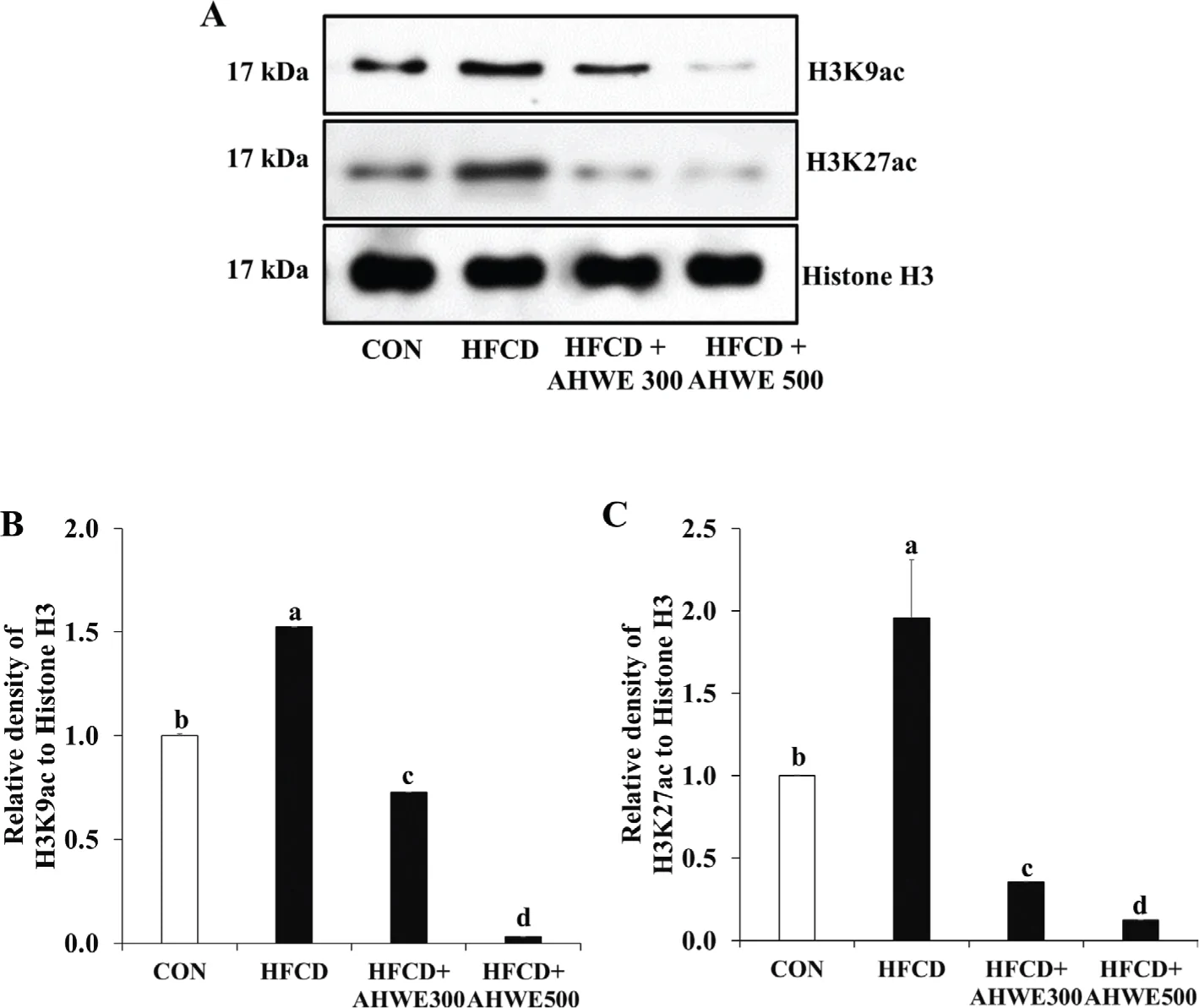
Effect of AHWE on histone acetylation in the liver of HFCD-fed mice. (A) Protein levels of acetylated H3K9 and H3K27 were measured by immunoblotting using histones extracted from liver tissue. (B–C) Equal protein loading was confirmed by stripping and reprobing the membranes for histone H3; AHWE: *Allium hookeri* water extract; CON: control group (AIN-93G diet containing 7% kcal from fat); HFCD: high-fat and high-cholesterol diet containing 60% kcal from fat and 1% cholesterol; HFCD + AHWE 300: HFCD supplemented with 300 mg/kg/day AHWE; HFCD + AHWE 500: HFCD supplemented with 500 mg/kg/day AHWE; H3K9ac: acetylation of histone H3 at lysine 9; H3K27ac: acetylation of histone H3 at lysine 27; qPCR: quantitative polymerase chain reaction. Data are expressed as mean ± SEM (*n* = 5). Different letters indicate significant differences (*P* < 0.05) as determined one-way ANOVA followed by Duncan’s multiple range test.

### AHWE supplementation restored the reduced Treg cell population and function in vivo

To regulate immune hyperactivity, increased secretion of anti-inflammatory cytokines, such as IL-10 and TGF-β, by Tregs is essential. It is well established that both the population and function of Treg cells decline during the progression of atherosclerosis (16). To examine the effect of AHWE on the functional foxp3-Treg population in HFCD-fed mice, we analyzed the proportion of CD4^+^CD25^+^Foxp3^+^ T cells in the spleen. Figure 9 illustrates the impact of AHWE supplementation on the population of CD4^+^CD25^+^ T cells (Fig. 9A) and CD4^+^CD25^+^Foxp3^+^ T cells (Fig. 9B) in HFCD-fed mice. As shown in Fig. 9C, the proportion of Tregs was reduced in the HFCD group compared to the CON group (*P* < 0.05). However, HFCD + AHWE 500 restored the Tregs population compared to the HFCD (*P* < 0.05).

**Fig. 9:**
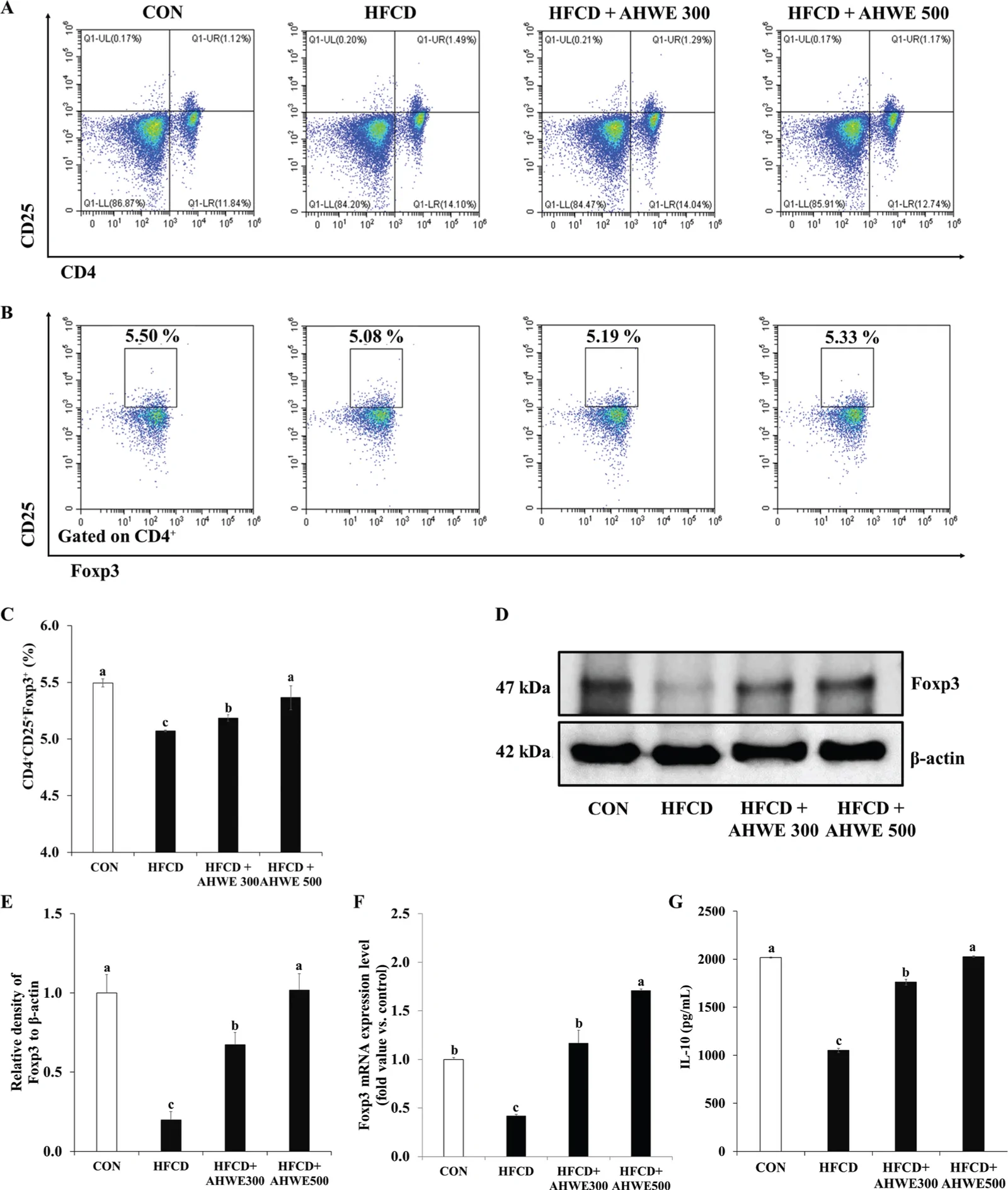
Effect of AHWE on the population and function of regulatory T cells in HFCD-fed mice. (A) Representative dot plots showing the proportion of CD4^+^CD25^+^T cells in the spleen of HFCD-fed mice supplemented with AHWE, analyzed by flow cytometry. (B) Representative dot plots showing the proportion of CD4^+^CD25^+^ Foxp3^+^ T cells in the spleen of HFCD-fed mice supplemented with AHWE, analyzed by flow cytometry. (C) Quantification of CD4^+^CD25^+^Foxp3^+^ T cells expressed as a percentage of total CD4^+^ T cell. (D) Protein levels of Foxp3 was measured by immunoblotting using whole-liver lysates. (E) Equal protein loading was confirmed by stripping and reprobing the membranes for β-actin. (F) mRNA expression of Foxp3 was analyzed using qPCR and quantified by the 2^–ΔΔCT^ method. Expression levels were normalized to β-actin. (G) The secretion levels of IL-10 in serum were measured using ELISA kit. AHWE: *Allium hookeri* water extract; CON: control group (AIN-93G diet containing 7% kcal from fat); HFCD: high-fat and high-cholesterol diet containing 60% kcal from fat and 1% cholesterol; HFCD + AHWE 300: HFCD supplemented with 300 mg/kg/day AHWE; HFCD + AHWE 500: HFCD supplemented with 500 mg/kg/day AHWE; Foxp3: forkhead box P3; IL-10: Interleukin-10. Data are expressed as mean ± SEM (*n* = 5). Different letters indicate significant differences (*P* < 0.05) as determined one-way ANOVA followed by Duncan’s multiple range test.

To confirm the functional expression level of Treg cells, we analyzed the level of Foxp3 expression and the secretion ability of the anti-inflammatory cytokine IL-10. Foxp3 protein expression was analyzed by immunoblotting using whole-spleen lysates. As shown in Fig. 9D–F, the expression level of Foxp3 was significantly reduced in the HFCD group relative to the CON group. However, AHWE supplementation significantly restored Foxp3 expression compared to the HFCD. Likewise, IL-10 secretion was diminished in the HFCD group, but was notably elevated in the AHWE supplementation group (Fig. 9G).

### AHWE treatment enhanced the immunoregulatory function of Treg cells in vitro

To further investigate how AHWE increases the population and function of Treg cells in mice, we isolated CD4^+^CD25^+^Foxp3^+^ T cells and cultured them in vitro. The results of this experiment are presented in Fig. 10. The purity of the isolated Tregs was confirmed to be over 70% (Fig. 10A and 10B). IL-2 is essential for Treg survival and function (33). As shown in Fig. 10C, no significant differences in the Treg population were observed between the groups with or without IL-2. However, AHWE treatment led to a dose-dependent increase in the Treg cell population (Fig. 10C and 10D). Similarly, cell viability increased in a dose-dependent manner following AHWE treatment (*P* < 0.01), as determined by flow cytometry (Fig. 10E). Moreover, IL-2 supplementation enhanced cell viability compared to the untreated group (*P* < 0.05). As shown in Fig. 10F, Foxp3 mRNA expression increased in a dose-dependent manner following AHWE treatment (*P* < 0.01) in vitro. PGRN can preserve Foxp3 expression in Tregs, maintain their regulatory function, and mitigate inflammation within atherosclerotic lesions (34). As shown in Fig. 10G, PGRN mRNA expression was increased at a high concentration (250 μg/mL) of AHWE (*P* < 0.05). As shown in Fig. 10H, the IL-10 levels were increased in a dose-dependent manner following AHWE treatment (*P* < 0.05).

**Fig. 10:**
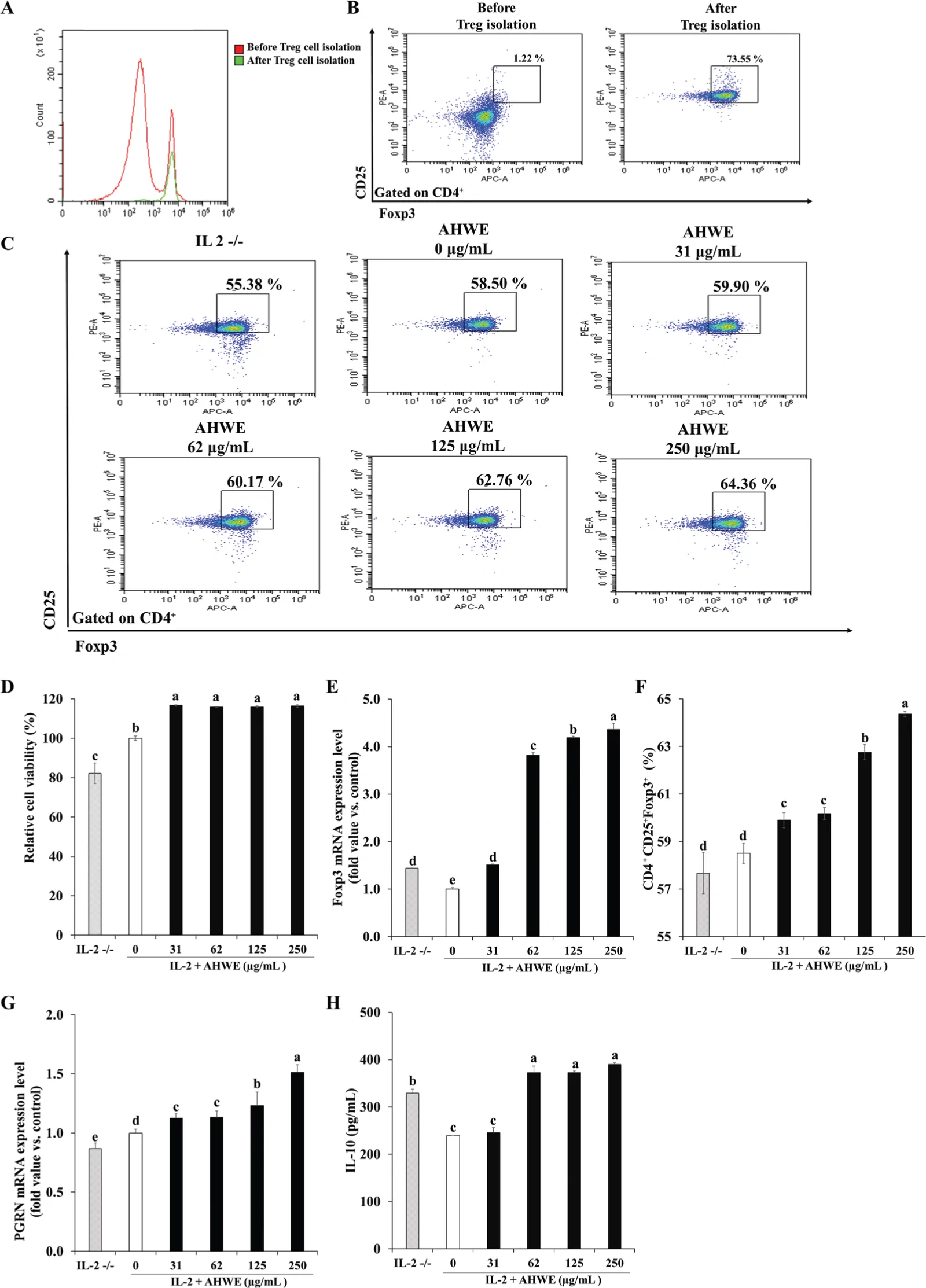
Effects of AHWE on the population and function of regulatory T cells *ex vivo*. (A) Histograms showing the populations of CD4^+^ T cells before and after Treg cell isolation. (B) Representative dot plots showing the population of CD4^+^CD25^+^Foxp3^+^ T cells before and after Treg cell isolation. (C) Representative dot plots of CD4^+^CD25^+^Foxp3^+^ T cells after 48 h cell culture under the following conditions: without IL-2, or with IL-2 supplemented with various concentrations of AHWE (31–250 µg/mL). (D) Quantification of CD4^+^CD25^+^Foxp3^+^ T cells expressed as a percentage of total CD4^+^ T cells. (E) cells were stained with a viability dye to distinguish live and dead cell populations. (F–G) mRNA expression of Foxp3 and PGRN were analyzed using qPCR and quantified by the 2^–ΔΔCT^ method. Expression levels were normalized to β-actin. (H) The secretion levels of IL-10 in the cell culture supernatant were measured using an ELISA kit. AHWE: *Allium hookeri* water extract; CD4: cluster of differentiation 4; CD25: interleukin-2 receptor alpha chain; Foxp3: forkhead box P3; IL-2: interleukin-2. Data are expressed as mean ± SEM (*n* = 2). Different letters indicate significant differences (*P* < 0.05) as determined by Duncan’s multiple range test.

## Discussion

*Allium hookeri* has been used as a food ingredient, and several studies have reported its potential biological activities, including anti-inflammatory and metabolic effects. However, evidence regarding its protective effects against atherosclerosis and the underlying mechanisms remains limited. In this study, we provide new evidence that AHWE may protect against HFCD-induced atherosclerotic changes through the coordinated regulation of lipid metabolism, inflammatory responses, and immune homeostasis. AHWE supplementation improved serum lipid profiles and insulin sensitivity and attenuated hepatic histological alterations and aortic thickening in HFCD-fed mice. These phenotypic improvements were accompanied by enhanced PPARγ/LXRα/ABCA1 signaling, reduced expression of the scavenger receptors CD36, SR-A1, and LOX-1, activation of AMPK with suppression of mTOR signaling, and attenuation of NF-κB-mediated inflammatory responses. In addition, AHWE increased the Treg population and IL-10 expression and altered histone acetylation. Collectively, these findings suggest that the beneficial effects of AHWE on atherosclerosis may involve coordinated improvements in lipid metabolism and inflammatory and immune responses.

Obesity-induced inflammation is a key driver of metabolic dysfunction, including insulin resistance and CVD development (35). In this study, an atherosclerosis model was established by feeding C57BL/6 mice an HFCD to induce obesity. Our findings showed that HFCD significantly increased body weight gain and tissue weight, accompanied by lipid accumulation in the liver and adipose tissue. These changes were effectively attenuated by AHWE supplementation. Similar effects on reducing body weight gain, adipose tissue mass, and hepatic lipid accumulation in high-fat diet-induced obese mice have been reported for other natural extracts, such as Carya cathayensis leaf extract and Artemisia capillaris extract (36, 37).

In the initial phase of atherosclerosis, serum LDL-C functions as a key marker of disease onset. When LDL-C levels are elevated, they are prone to oxidative modification, and this oxidized form of LDL-C is considered a critical contributor to both the initiation and progression of coronary atherosclerosis (38). Our study demonstrated an abnormal lipid profile in the HFCD group compared to the other groups, as well as elevated fasting blood glucose and elevated insulin levels. AHWE supplementation significantly reduced serum TG, TC, and LDL-C levels while also increasing HDL-C levels in HFCD-fed mice. It also improved insulin sensitivity. Similarly, a previous study investigating an extract from a commonly consumed food ingredient reported that bitter melon extract supplementation improved glucose tolerance and insulin sensitivity while reducing serum TG and LDL-C levels in HFD-fed mice (39). The beneficial effects of AHWE may be associated, at least in part, with its bioactive organosulfur compounds. *Allium hookeri* contains various organosulfur compounds, including cycloalliin, which has been reported as one of its major bioactive constituents (32). In our previous study, AHWE and cycloalliin inhibited foam cell formation by promoting the PPARγ/LXRα/ABCA1-mediated cholesterol efflux pathway and suppressing scavenger receptor expression and inflammatory responses (36). These previous findings are consistent with the present in vivo results, in which AHWE enhanced cholesterol efflux-associated signaling, reduced the expression of CD36, SR-A1, and LOX-1, and attenuated inflammatory responses. Thus, cycloalliin may contribute, at least in part, to the anti-atherosclerotic effects of AHWE observed in this study. Taken together, these findings suggest that AHWE supplementation may exert protective effects against atherosclerosis by improving hepatic lipid metabolism and insulin sensitivity in HFCD-fed mice.

Fatty liver is closely associated with CVD, and hepatic lipid accumulation contributes to metabolic dysfunction, including dyslipidemia and insulin resistance (40). In addition, individuals with fatty liver have an increased risk of cardiovascular events, such as myocardial infarction and stroke (40). Atherosclerosis is characterized by the accumulation of lipid-rich plaques within the arterial wall, initiated by cholesterol deposition in the vascular intima (41). Our findings show that HFCD-fed mice exhibited marked hepatic vacuolation consistent with lipid accumulation, as observed by H&E staining, along with increased aortic intima–media thickness, indicating the progression of atherosclerotic changes. These histological alterations were substantially ameliorated by AHWE supplementation, suggesting a protective role against atherogenesis. This is consistent with a previous report demonstrating that dietary lycopene reduced aortic plaque burden and intima–media thickness in HFD-fed ApoE knockout mice, accompanied by histologically verified improvements in vascular structure and atherosclerotic lesion scores (42). These findings align with previous research emphasizing that fatty liver is not merely a hepatic disorder but a systemic condition with profound implications for cardiovascular health (40). However, lipid-specific staining, hepatic lipid content, and histological steatosis scores were not assessed in this study. Therefore, the H&E findings should be interpreted as morphological evidence consistent with hepatic lipid accumulation rather than as a direct assessment of hepatic steatosis. Further studies using lipid-specific staining and quantitative analysis of hepatic lipid content are warranted to confirm the effects of AHWE on hepatic lipid accumulation.

Plaque formation is accelerated by dysregulated cholesterol metabolism and enhanced inflammatory responses. Extensive in vivo evidence suggests that consumption of a high-fat diet promotes hepatic lipid accumulation and accelerates atherosclerosis progression (43). Among the key mediators of lipid uptake are scavenger receptors such as CD36, SR-A1, and LOX-1, which facilitate cholesterol influx into cells (6, 7, 44). In particular, CD36 deficiency has been shown to inhibit plaque formation in apoE–/– mice (44). In this study, expression of CD36, SR-A1, and LOX-1 was markedly increased in the HFCD group, whereas AHWE supplementation significantly downregulated their expression. The reverse cholesterol transport pathway plays a crucial role in maintaining cholesterol homeostasis by removing excess cholesterol from peripheral tissues and circulation, thereby preventing atherosclerotic lesion development in the aorta (45). Within this pathway, ABCA1 serves as a central regulator by facilitating the transfer of intracellular cholesterol to apoA1, a critical step in HDL formation (46) PPARγ promotes ABCA1-mediated cholesterol efflux through upregulation of LXR-α expression, while LXR-α independently functions as a key transcriptional regulator of ABCA1 (8). Accordingly, the PPARγ–LXR-α–ABCA1 signaling cascade is considered essential for maintaining cholesterol homeostasis and has emerged as a therapeutic target for atherosclerosis. Natural compounds including quercetin, curcumin, and sulforaphane have been reported to enhance cholesterol efflux (47). Similar to previous studies, AHWE supplementation activated the reverse cholesterol transport pathway by upregulating PPARγ, LXR-α, and ABCA1, which likely contributed to improving dysregulated cholesterol metabolism (48). Notably, PPARγ expression was elevated in both the HFCD and HFCD + AHWE groups compared with the CON group, consistent with previous findings that PPARγ activation enhances cholesterol efflux and promotes hepatic clearance of excess cellular cholesterol (24). Importantly, AHWE supplementation improved serum lipid profiles and attenuated hepatic vacuolar changes and aortic wall thickening in HFCD-fed mice. These biochemical and histological improvements were accompanied by reduced expression of the scavenger receptors CD36, SR-A1, and LOX-1, which are involved in lipid uptake, together with enhanced PPARγ/LXR-α/ABCA1 signaling associated with cholesterol efflux. Thus, the observed improvements in lipid-related biochemical and histological parameters may be associated with reduced lipid uptake and enhanced cholesterol efflux through coordinated regulation of these pathways.

Activation of AMPK suppresses lipid and cholesterol synthesis by phosphorylating key anabolic enzymes, including acetyl-CoA carboxylase (ACC) and HMG-CoA reductase (HMGCR), thereby reducing lipid accumulation (49). In contrast, chronic lipid overload during obesity can impair AMPK activation while enhancing mTOR signaling, which promotes adipogenesis and cholesterol synthesis and ultimately contributes to lipid accumulation (50). Previous studies have demonstrated that natural compounds alleviate metabolic dysfunction through modulation of the AMPK/mTOR signaling pathway. For example, pectic bee pollen polysaccharide extracted from Rosa rugosa improved hepatic lipid accumulation and insulin resistance in HFD-induced obese mice by regulating AMPK/mTOR signaling (50). Similarly, red ginseng and green tea extract have been reported to activate AMPK, reduce lipid levels, and attenuate atherosclerosis progression in murine models (51). Beyond its role in lipid metabolism, AMPK is also recognized as an important regulator of inflammatory responses. AMPK activation suppresses pro-inflammatory signaling cascades and reduces the accumulation of lipid intermediates that can trigger immune activation, whereas pro-inflammatory mediators have been shown to inhibit AMPK activation and impair lipid oxidation (52). Chronic inflammation is closely associated with the progression of obesity and atherosclerosis, and inflammatory cytokines are critically involved in the development and destabilization of atherosclerotic plaques (13). In particular, IL-6, TNF-α, COX-2, and NF-κB are key mediators implicated in atherosclerosis progression (53). In this study, HFCD reduced AMPK activation while increasing mTOR signaling and inflammatory mediator expression. In contrast, AHWE supplementation restored AMPK activation, suppressed mTOR signaling, and significantly reduced the expression of IL-6, TNF-α, COX-2, and NF-κB in HFCD-fed mice. These findings are consistent with our in vitro results and suggest that AHWE may alleviate lipid accumulation and inflammatory responses through modulation of the AMPK/mTOR/NF-κB signaling pathway.

Persistent low-grade systemic inflammation caused by prolonged immune activation plays a critical role in the development of CVDs, including atherosclerosis (54). In atherosclerosis, immune imbalance characterized by increased pro-inflammatory T cell responses and impaired Treg function plays a critical role in plaque progression (18). While Th1 cells promote vascular inflammation through the secretion of TNF-α and IFN-γ, Tregs maintain immune homeostasis and exert immunosuppressive effects through the production of anti-inflammatory cytokines such as TGF-β and IL-10 (55). However, chronic inflammatory conditions impair Treg function and enhance inflammatory Th1 and Th17 responses, thereby accelerating atherosclerotic progression and immune dysregulation (56). Therefore, strategies aimed at restoring the immunoregulatory function of Tregs have emerged as promising therapeutic approaches for mitigating atherosclerosis. Our study demonstrated that HFCD-fed mice exhibited a marked decrease in Treg populations, along with reduced Foxp3 expression, a key transcription factor required for Treg maintenance. However, AHWE supplementation restored Treg populations and function by significantly increasing Foxp3 expression and IL-10 secretion, suggesting a protective effect against inflammation-driven Treg dysfunction. Several studies have indicated that dietary interventions can mitigate the detrimental effects of HFD on immune regulation, particularly by enhancing the population of Treg cells. For example, Camacho-Muñoz et al. reported that omega-3 polyunsaturated fatty acids supplementation in a Western diet model significantly increased the proportion of CD4+Foxp3+ Tregs in lymphoid organs by attenuating ceramide-induced T cell dysfunction (55). Lewis et al. demonstrated that dietary blueberry supplementation improved T cell-mediated immune function in HFD-induced obese mice, partially restoring T cell proliferation and cytokine production compared to the HFD-only group (57). Consistent with these previous studies, our results indicate that AHWE may preserve Treg populations by modulating inflammatory responses in HFCD-fed mice. IL-2 is a crucial cytokine for Treg survival, proliferation, and function, which stabilizes Foxp3 expression and maintains Treg suppressive capacity (33, 58). In our study, regardless of IL-2 supplementation, AHWE treatment dose-dependently enhanced Treg population and viability, accompanied by increased expression of Foxp3 and PGRN, as well as elevated IL-10 secretion. These findings suggest that AHWE may enhance Treg population and function, in part by promoting anti-inflammatory gene expression and cell survival.

Additionally, emerging evidence suggests that chronic inflammation and metabolic dysfunction are closely associated with epigenetic alterations involved in atherosclerosis progression. A recent study has demonstrated that in obese conditions, H3K9ac is significantly elevated in the liver, which disrupts normal hepatic function by impairing lipid metabolism, ultimately contributing to liver dysfunction and the progression of metabolic disorders (20). According to Cordero-Herrera et al., (–)-epicatechin – the main flavanol in cocoa – was effective in attenuating H3K9ac induced by high glucose (59). H3K9ac has been shown to enhance the activity of MMPs, which degrade extracellular matrix components and thereby contribute to plaque instability and rupture during atherosclerotic progression. Moreover, both H3K9ac and H3K27ac directly upregulate the expression of the pro-inflammatory cytokine IL-6, a critical mediator in plaque formation and progression. Furthermore, H3K27ac has been reported to increase reactive oxygen species levels, leading to oxidative stress, which further accelerates atherosclerotic development. In our study, HFCD increased H3K9ac and H3K27ac in hepatic tissue, whereas AHWE supplementation modulated H3K9ac and H3K27ac levels. These findings suggest that AHWE supplementation effectively mitigates epigenetic alterations that exert detrimental effects on lesion progression by reducing H3K9ac and H3K27ac, suggesting protective effects against epigenetic alterations associated with atherosclerosis progression. Taken together, atherosclerosis is driven by interconnected metabolic, lipid-related, and inflammatory processes that contribute to macrophage lipid accumulation, foam cell formation, and plaque development. In this study, AHWE modulated multiple pathways associated with these processes, including reduced expression of the scavenger receptors CD36, SR-A1, and LOX-1, enhanced PPARγ/LXRα/ABCA1-mediated cholesterol efflux signaling, suppression of NF-κB-mediated inflammatory responses, and enhancement of Treg/IL-10-related immune regulation. These coordinated changes suggest that AHWE may improve cholesterol homeostasis and attenuate the pro-inflammatory environment associated with early atherogenesis. These effects may, at least in part, be attributable to bioactive constituents of *A. hookeri*, particularly organosulfur compounds such as cycloalliin. However, atherosclerotic plaque burden and foam cell formation were not directly quantified in this study, and the contribution of individual bioactive compounds to the observed effects was not directly examined. Therefore, further studies using direct quantitative assessments of atherosclerotic lesions and foam cell formation, together with isolated or standardized bioactive compounds, are warranted to confirm the anti-atherosclerotic mechanisms of AHWE and identify the specific constituents responsible for these effects.

## Conclusions

AHWE supplementation attenuated body and tissue weight gain and improved metabolic parameters in C57BL/6 mice fed the HFCD. Hepatic lipid accumulation and vascular endothelial dysfunction in the aorta were also alleviated by AHWE supplementation. Mechanistically, AHWE promoted cholesterol efflux through upregulation of PPARγ, LXR-α, and ABCA1, while suppressing lipid accumulation by downregulating SR-A1, CD36, and LOX-1. In addition, AHWE restored abnormal lipid metabolism by enhancing AMPK activation and inhibiting mTOR signaling. AHWE supplementation also reduced inflammatory responses by suppressing NF-κB, TNF-α, COX-2, and IL-6. Furthermore, AHWE increased Treg populations and enhanced their anti-inflammatory function, as indicated by elevated Foxp3 and IL-10 expression. Consistent with these findings, ex vivo analysis confirmed the Treg-associated anti-inflammatory effects of AHWE and increased PGRN expression following AHWE treatment. Moreover, AHWE supplementation reduced H3K9ac and H3K27ac levels, suggesting protective effects against epigenetic alterations associated with atherosclerosis progression. Taken together, these findings suggest that AHWE supplementation may alleviate obesity-induced atherosclerosis in HFCD-fed mice by improving cholesterol homeostasis and lipid metabolism, suppressing inflammatory responses, enhancing Treg-mediated immunoregulation, and reducing histone acetylation.
